# Comparative population genomics reveal stark differences in the genomic structure and drivers of isolation for two sky island bumble bees

**DOI:** 10.1098/rspb.2026.1217

**Published:** 2026-10-07

**Authors:** Laura J. Jones, Lauren C. Ponisio, Shalene Jha

**Affiliations:** ^1^ Department of Integrative Biology, The University of Texas at Austin, Austin, TX, USA; ^2^ Institute of Ecology and Evolution, University of Oregon, Eugene, OR, USA; ^3^ Lady Bird Johnson Wildflower Center, Austin, TX, USA

**Keywords:** *Bombus*, sky island, gene flow, isolation, resistance, single nucleotide polymorphism

## Abstract

Genetic diversity is critical for species’ persistence and for preserving biodiversity. Regions with extreme elevation gradients, including ‘sky island’ systems, are predicted to restrict gene flow owing to dispersal barriers between high-elevation habitat patches (isolation by resistance, IBR) and the sheer distances between patches (isolation by distance, IBD). However, the drivers of genomic structure for sky island species remain poorly characterized. We evaluated the effects of IBR and IBD on the genomic structure of two co-occurring bumblebees, *Bombus centralis* and *Bombus huntii*, in the Cordilleran and Madrean sky islands using single-nucleotide polymorphism data. These species share similar sizes and diet breadths, but *B. centralis* is strongly associated with high-elevation forest and grasslands, representing only a subset of habitats used by *B. huntii*. We found that both species are experiencing moderate to high levels of inbreeding, but that *B. centralis* populations are highly differentiated, while *B. huntii* shows little genomic structure. IBR owing to land cover and elevation was evident for both, with support for IBD found only in *B. huntii*. Our study highlights the risk of isolation in sky island systems and demonstrates how small differences in ecological niche space can lead to dramatically different genomic structure.

## 1. Introduction

Conserving genetic diversity is essential for population-level resilience and species-level biodiversity maintenance in the face of global change [1]. Ecological isolation owing to landscape composition-based barriers (isolation by resistance, IBR) or geographic distance-based barriers (isolation by distance, IBD) can lead to reductions in gene flow among populations, increasing the risk of inbreeding depression and, ultimately, local extinction [2]. Therefore, it is crucial to understand patterns of species-level genomic structure, which can characterize both within- and cross-population demographic processes, such as coancestry and inbreeding, while also shedding light on potential dispersal barriers experienced by different organisms. Past studies have demonstrated that genomic structuring can capture distinct life-history traits and habitat needs, even for closely related species [3–5], and can reveal the specific contemporary and historical processes that shape population divergence [6–9]. Multi-species comparative studies are especially helpful in isolating distinct drivers of population structure [10,11], which is particularly important for understanding and advancing the conservation of small-bodied organisms, such as insects, that are especially sensitive to climate and habitat change [12–14]. Thus, assessing the qualities of a landscape that differentially impact gene flow for co-occurring, small-bodied species can provide key insight into the specific traits underlying an organism's sensitivity to global change and could further allow for the identification of at-risk species in the context of future shifts in climate or land use.

Eusocial bee species, including many within the genus *Bombus* (i.e. bumble bees), have characteristics that render them susceptible to movement barriers but also capable of long-distance dispersal, providing a unique opportunity to investigate species-specific drivers of gene flow across landscapes. First, the reproductive needs and foraging strategy dictated by eusociality may limit gene flow because, once a bumble bee colony is established, the workers act as central-place foragers, which results in the prioritization of nest site establishment near abundant forage above all else [15]. Indeed, there is evidence for many bumble bee species, including *Bombus flavifrons, B. hortorum, B. lapidarius, B. pascuorum* and *B. vosnesenskii*, that queens exhibit high philopatry, with average dispersal ranging only 1–5 km away from their overwintering sites [16–21]. By contrast, the range-expanding, high floral resource generalists *B. ruderatus* and *B. terrestris* have been estimated to disperse as far as 15–95 km per year after their introductions in Chile [15,22], highlighting the context-dependency of dispersal capacity in this genus. Further, male dispersal is also an important factor mediating inbreeding; past work indicates that bumble bee males often aggregate near the nest sites of other colonies and may perform hill-topping behaviour across various habitats [23,24], suggesting that there are mechanisms in place to avoid within-colony mating regardless of the dispersal capacity of new queens [25,26]. Given the evidence that queen dispersal is impacted by resource availability during nest site establishment [17,21,22,27,28] and that males may respond to habitat features as they scout for queens [24], it is likely that both raw distance and land cover quality have large impacts on the movement of bumble bee reproductives. Assessing the population structure and inbreeding levels of *Bombus* species within populations, over large distances and across heterogeneous habitats can help elucidate the spatial and landscape features that most influence dispersal and gene flow.

Further, past population genomic work suggests that bumble bees may be a model species for understanding IBD and IBR impacts on climate-sensitive species given their responsiveness to fine-scale habitat features. Specifically, even for bumble bees with low regional differentiation, local-scale changes to landscape composition, mostly driven by human land-use, including urbanization, agriculturalization and deforestation, have indeed been identified as significant drivers of IBR [11,19,29–31], as seen in other bee systems [3,32,33]. While these patterns are well-documented in highly modified landscapes, ecosystems with little human-induced land-use change but substantial natural variation in habitat, such as high elevation forests and meadows, show less conclusive patterns of IBR despite consistent support for IBD in bumble bees. For example, in the northern extent of their distribution, gene flow for the bumble bee *B. huntii* is found to be uninhibited by environmental heterogeneity (i.e. no support for IBR), and what little structuring is present is explained by IBD alone [34]. By contrast, both Lozier and colleagues [35] and Jackson and colleagues [11] found strong regional clustering in a different species, *B. vancouverensis* (formerly *B. bifarius*) [36], explained by both IBR and IBD, with IBR receiving stronger support. Overall, these past studies reveal that bumble bees may differ in their response to distance or land cover resistance, but it remains unknown which landscape features or species traits drive these differences, particularly in minimally managed, higher-elevation habitats. In addition, to date, most work interrogating the role of land cover resistance in mediating gene flow for bees has used broad land cover categories (i.e. forest, grassland, urban and crop) with binary resistance assignments and has relied on limited human knowledge of habitat use to determine resistance values. However, montane regions can exhibit vast differences in vegetative community composition, even among habitats with similar land cover classifications [37]. Thus, to capture the wide variability in habitat suitability and resistance found within heterogeneous landscapes, such as montane systems, it is particularly important to consider a finer classification of land cover classes.

Sky island ecosystems offer a robust model system in which to investigate bumble bee population genomic structuring and inbreeding patterns across distinct co-occurring species in the presence of both elevation and natural land cover variation. ‘Sky islands’ are characterized by high turnover in biotic communities moving up in elevation, where temperate forest or grassland refugia (i.e. ‘islands’) present at high elevations are surrounded by extensive, low-elevation deserts (i.e. ‘sea’) [38]. For many high-elevation taxa, repeated Pleistocene glaciations allowed for gene flow between populations occupying different mountains as their habitats shifted to lower elevations; each glaciation event was followed by warming that increased divergence, eventually forming the current temperate ‘sky islands’ [39]. These repeated patterns of historic isolation are evident in the high genomic structuring observed in species currently restricted to these sky island refugia [40–42], particularly in insects [10,43–46], and are associated with high inbreeding and risk of inbreeding depression [40,42,47,48]. Some highly mobile species, however, most notably birds, are found to have less structuring, suggesting that flight may aid dispersal between high-elevation sites [47,49]. Despite the past work in this iconic system, it is unknown to what extent smaller organisms with more limited flight capacity can disperse between isolated, high-elevation habitats. While bumble bees can maintain regional gene flow across some elevational gradients [11,30,31,34], the high divergence found for other insects in sky islands suggests that bumble bees may also be more dispersal-limited in these systems. Notably, because sky islands are characterized by drastic elevation shifts and vegetation turnover across short distances, corridors with suitable habitat for dispersal are often limited or non-existent between mountains [39], unless a species exhibits a high tolerance for heterogeneous habitat. Given their propensity for resource generalism and variation in elevational niche, bumble bees offer a unique system for comparative work to evaluate dispersal limitation in sky island complexes.

In this study, we investigated the effects of IBD (distance) and IBR (land cover, elevation) on the genomic structuring and inbreeding levels of two co-occurring bumble bee species, *B. centralis* and *B. huntii*, in the Cordilleran and Madrean sky islands. These species were selected because they share many relevant traits that impact establishment and dispersal capacity (e.g. similar in size, nesting strategy, diet breadth and sociality) [50], but vary in their elevation and land cover-based habitat niches, allowing us to compare the relative impacts of distance, elevation and land cover as gene-flow barriers, while holding constant other life history traits. Specifically, *B. centralis* is found only at higher elevations [51] and is documented in temperate forest and grassland habitats [50,52], while *B. huntii* occupies these landscapes in addition to arid habitats, including desert scrubland [50–52]. We expect that *B. huntii* will have higher genetic connectivity and lower levels of inbreeding owing to its occupation of lower-elevation habitats and its greater tolerance for arid environments, and that only distance will impede gene flow between its populations (supporting IBD). By contrast, we expect that *B. centralis* will exhibit high genomic structuring and high levels of inbreeding owing to its isolation at higher elevations, and we predict that resistance owing to changes in elevation and land cover across the landscape will better explain genomic structure (support for IBR) than distance alone (IBD). Overall, this cross-species comparison contributes to our broader understanding of gene flow in highly heterogeneous habitats and gives insight into potential inbreeding risks and, thus, conservation priorities for vulnerable, small-bodied organisms in dynamic high-elevation ecosystems.

## 2. Methods

### (a) Site selection and sampling

We sampled eight meadows (average distance of 89.09 ± 50.45 km between sites; average elevation of 2901 ± 143 m) in the years 2012, 2017, 2018, 2021 and 2022 across the Cordilleran and Madrean sky islands located in the southwestern USA ( figure 1). This site sampling number is typical for remote, montane ecosystems, which are largely composed of private and protected lands that are difficult to access [5,48,49]. We further enforced a minimum threshold for inclusion in this study of five females collected for a species at that site, a per-site sampling level that is sufficient for characterizing population structure [49,53], resulting in 151 *B. centralis* and 97 *B. huntii* individuals (supplementary material, table S1). At each site, active netting was performed for 10 hours during each sampling year. Collections were performed on sunny days with air temperatures between 17 and 24°C and wind speeds below 2.5 m s^–1^. All netted samples collected in 2012 (*n* = 5 *B. centralis,* 10 *B. huntii*) and 2017 (*n* = 25 *B. centralis,* 25 *B. huntii*) were transferred to a cyanide jar at room temperature. Eight samples in 2017 were collected via a combination of pan traps (*n* = 2) and blue vane traps (*n* = 6) [54] and stored in 90% ethanol. For pan traps, a set of one blue, white and yellow trap was filled with 0.01% soap solution and placed at five different locations within each site for 4.5 hours. For blue vane traps, three traps were filled with propylene glycol and placed at three different locations within each site for 24 hours. All netted and trapped samples were identified, pinned and placed in long-term storage at room temperature. In 2018, 2021 and 2022, our netting protocol additionally included sterile collection to reduce microbial contamination of bee individuals for later use in intestinal parasite and microbiome screening [55]. Specifically, we sterilized gloves and netting equipment with 10% bleach followed by 70% ethanol between each sampling round. Samples in 2018 (*n* = 34 *B. centralis,* 22 *B. huntii*), 2021 (*n* = 62 *B. centralis,* 20 *B. huntii*) and 2022 (*n* = 25 *B. centralis,* 20 *B. huntii*) were collected on dry ice in the field, identified and stored at –80°C.

**Figure 1.**
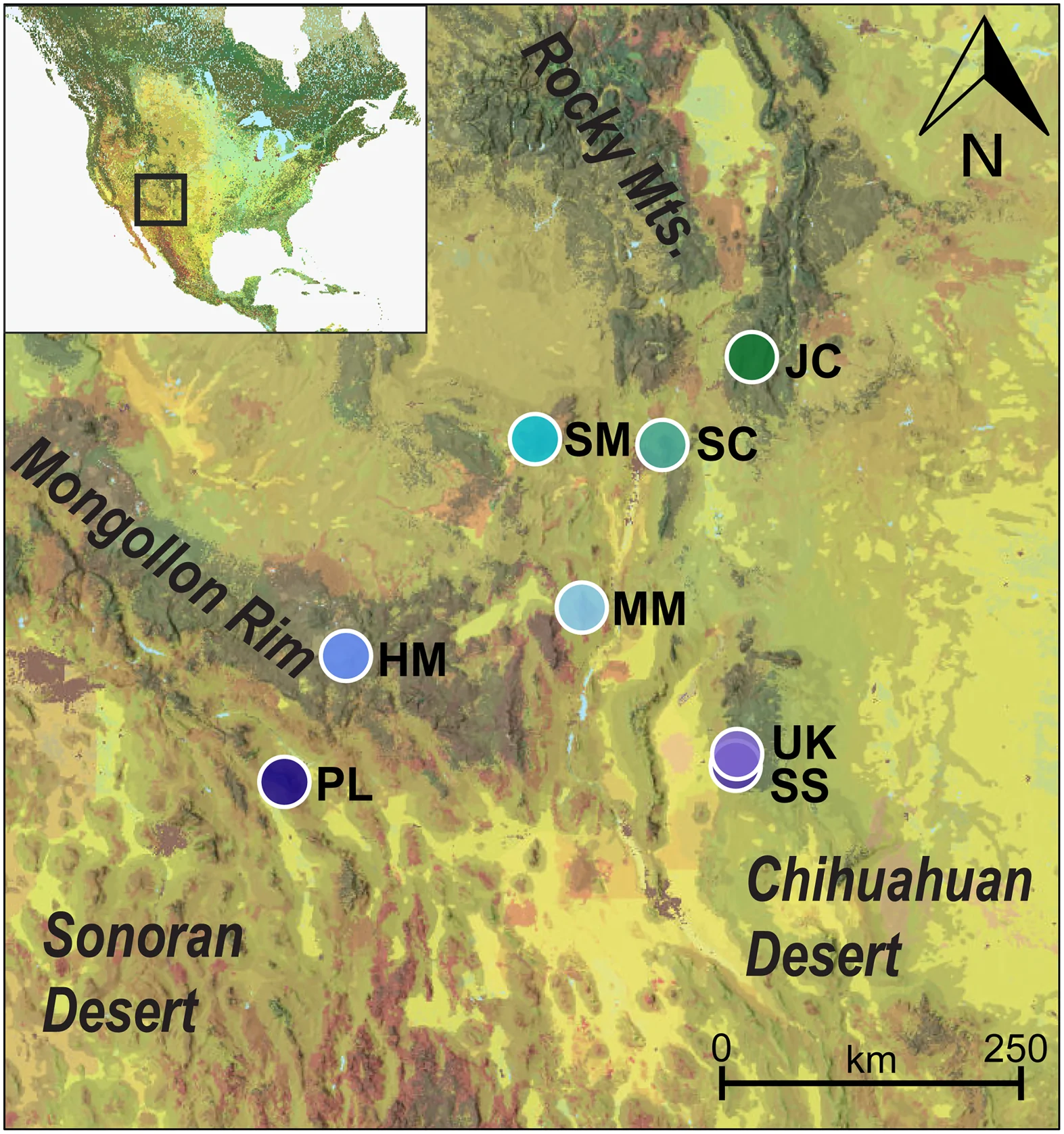
Sites sampled to evaluate the genomic structure of *B. centralis* and *B. huntii* in the Cordilleran and Madrean sky islands in Arizona and New Mexico, USA (black box in inset indicates study region within North America). Site code is indicated next to points (see supplementary material, table S1 for details on mountain range, meadow and bee species sampled at each site). The base topographic layer used is the 2015 World Ecological Land Units Map (USGS, Esri). The map was generated using ArcGIS Online.

### (b) DNA extraction

Gut tissue was dissected and stored at –80°C for 96 *B. centralis* and 40 *B. huntii* collected in 2018, 2021 and 2022 to perform targeted screening of intestinal parasites and microbiome [55], and this tissue was then used for our nextRAD sequencing. Abdomen tissue (i.e. all abdominal contents and exoskeleton) was dissected from the remaining 55 *B. centralis* and 57 *B. huntii*, and both sets of samples (*n* = 248) were combined to assess population genomic structure (supplementary material, table S1). DNA was isolated using the Qiagen DNeasy 96 Blood & Tissue Kit (cat. no. 69581) followed by RNase treatment. We quantified DNA using the Introvigen™ Qubit™ dsDNA Quantification Assay Kit (cat. no. Q32851) and a Qubit 3.0 Fluorometer, and we assessed RNA or solvent contamination using a Thermo Scientific NanoDrop Spectrophotometer. Genomic DNA was visualized on 1% agarose gel to assess quality.

### (c) NextRAD sequencing and genotyping

NextRAD library preparation and sequencing were performed by SNPsaurus using 5 ng genomic DNA per sample [56] (see supplementary material; BBMap tools, v. 39.52; http://sourceforge.net/projects/bbmap/). All reads were mapped to the *B. huntii* reference genome (GCA_024542735.1; NCBI GenBank) with an alignment identity threshold of 90% using bbmap (BBMap tools). We separated samples by species and used callvariants for genotype calling with a minor allele frequency (MAF) of 5% (BBMap tools) to distinguish true polymorphisms from sequencing artefacts. We assigned any genotype with a depth lower than 8% as missing. After genotyping, we removed any single nucleotide polymorphisms (SNPs) with an average quality score below 30% and an average depth below 5%, and all indels, using BCFtools (v. 1.22) [57]. Next, we removed loci with more than 50% missing data and then reapplied frequency-based filters (MAF = 5%). We allowed for 50% missing data given evidence that there is no systematic effect of this threshold on population genomics statistics or demographic parameters if adequate quality filters are applied [58]. Lastly, any loci with greater than 60% heterozygosity were removed using VCFtools (v. 4.2) [59] as per [11,60]. We used the Hierfstat package (v. 0.5-11) [61] in R to calculate heterozygosity per locus (final loci totals after filtering: *B. centralis* = 5064 and *B. huntii* = 3761). After SNP filtering, we removed any individuals missing more than 80% of data using VCFtools, resulting in 121 *B. centralis* and 80 *B. huntii* individuals. We employed this 80% cut-off given evidence that missingness at similar levels does not impact population structure analyses [62–64] (see supplementary material, table S4 for population-level assessment of *H_O_* and *F_IS_*).

### (d) Kinship and colony assignment

We identified probable sibling groups using the pairwise relationship function *relatedness2* in VCFtools [65]. This function implements the KING-robust algorithm that provides a relatedness score (*Φ*_*ij*_) between 0 (not related) and 0.5 (identical twin or replicate sample) using only the SNP data for the individuals being compared. We identified pairs with a relatedness score greater than 0.2 as probable kin (supplementary material, table S2). This cut-off is supported in other *Bombus* spp. studies [11,60]. For the following analyses, we removed all but one individual within kin groups based on mean sequencing depth to maintain one representative per colony. Frequency-based filters (MAF = 5%) were re-applied to remove potential artefacts in the final dataset. The final sample totals after kin removal were 120 *B. centralis* and 77 *B. huntii,* similar in total sample size to past bumble bee genomic studies [29,31,66].

### (e) Genomic structure and gene diversity

We evaluated the genomic structure for *B. centralis* and *B. huntii* independently. First, we confirmed that site missingness and individual missingness were less than 50% and 80%, respectively, using the package Hierfstat in R. We calculated overall fixation indices (*F_ST_* and *D_EST_*), inbreeding coefficient (*F_IS_*), within-population heterozygosity (*H_O_*), population-level gene diversity (*H_S_*), species-level gene diversity (*H_T_*) and gene diversity among samples (*D_ST_*) using the basic.stats function (supplementary material, table S3). We tested the significance of overall *F_ST_* and *F_IS_* with a permutation test in which population code was randomly reassigned among individuals over 1000 permutations to generate null distributions. We then estimated population-level *F_IS_*, *H_O_* and *H_S_* (supplementary material, table S4) and calculated pairwise *F_ST_* between populations (supplementary material, table S5). We determined private allele counts per population and normalized by sample size, using the function private_alleles in the package poppr (v. 2.9.8) [67]. To assess population structure, we performed discriminant analysis of principal components (DAPC) using the package adegent (v. 2.1.11) [68]. We calculated the optimal alpha score using the function optim.a.score to inform the number of principal components to retain in the DAPC (seven for *B. centralis*, 15 for *B. huntii*). Additionally, we calculated the goodness of fit (Bayesian information criterion) for successive k-means using the find.clusters function; this function determines the number of distinct genetic clusters for each species (supplementary material, figure S1).

To evaluate recent coancestry among individuals, we used RADpainter to infer a coancestry matrix using haplotype-based inference and fineRADstructure to assign individuals to inferred genetic clusters using Bayesian inference separately for *B. centralis* and *B. huntii* [69]. We used a burn-in of 100 000 iterations, followed by 100 000 iterations for Markov chain Monte Carlo with a thinning interval of 1000. We built the tree using posterior population-assignment probabilities with default parameters in fineRADstructure (supplementary material, figure S2).

### (f) Drivers of genetic differentiation (isolation by distance/isolation by resistance)

We used pairwise *F_ST_* values to investigate if geographic distance, elevation and land cover influenced patterns of genetic differentiation in *B. centralis* and *B. huntii*. Specifically, we calculated pairwise *F_ST_* (function pairwise.neifst, package Hierfstat) separately for each species, following Weir & Cockerham [70], and tested for significance via bootstrapping over loci of pairwise *F_ST_* with 1000 bootstraps to obtain confidence intervals (function boot.ppfst, package Hierfstat) (supplementary material, table S5). We collected elevation data for the most recent sample year (2022) at a resolution of one arc-second (approx. 30 m) from the National Elevation Dataset (U.S. Geological Survey, EROS Data Center). We also collected land cover data for the year 2020 (within the sampled year range) at a 30 m resolution from the North American Land Change Monitoring System (Multi-Resolution Land Characteristics Consortium). Both rasters were aggregated to 300 m resolution (function aggregate; package terra, v. 1.8-70) (supplementary material, figure S3).

We optimized elevation and land cover resistances ranging from 1 (low resistance) to 1000 (high resistance) using a genetic algorithm (package resistanceGA, v. 4.2-10) [71] to comprehensively evaluate the parameter space of resistance surfaces and to avoid bias arising from traditional approaches that assign resistance values using *a priori* or expert knowledge of habitat use [30–32,72]. ResistanceGA conducts iterative transformations (continuous) or reclassifications (categorical) of environmental data into resistance values to calculate pairwise resistance distances between populations, and fits linear mixed effects models after each iteration to compare and identify the resistance surface which best supports the genetic differentiation between populations [71]. This optimization process continues until 25 consecutive iterations do not improve the model fit. We optimized resistance surfaces independently per species (function SS_optim) using Circuitscape-based optimization (CS.prep) and default conditions in resistanceGA (figure 2; supplementary material, table S7). For geographic distances, we calculated the Euclidean distance between each pair of populations. We fit generalized least squares (GLS) models (function gls; package nlme, v. 3.1-163) [73] with a maximum likelihood population effects (MLPE) correlation structure (function corMLPE; package corMLPE, v. 0.0.3) [74] to account for non-independence among pairwise data. All predictor variables were correlated (variance inflation factor (VIF) greater than 5) for both species, so we fit separate elevation (IBR), land cover (IBR) and distance (IBD) models and used corrected Akaike information criterion (function AICc; package MuMIn, v. 1.47.5) [75] to compare model fits, similar to past studies [31,32,76,77]. We further tested the potential roles of climate-based drivers of isolation using rasters collected at a coarser resolution (1 km; DAYMET) and found that temperature performed similarly to elevation (per AICc), while precipitation was not a strong predictor of differentiation for either species (supplementary material, figures S3 and S4, tables S7 and S8).

## 3. Results

### (a) Relatedness

After filtering, we produced genotypes for 121 *B. centralis* and 80 *B. huntii* in the Cordilleran and Madrean sky islands region, totalling 5064 SNPs and 3761 SNPs, respectively. We found one sibling group for *B. centralis* at one site in the year 2018 (individual *i* related to *j*1 and *j*2; *Φ*_*ij*1_ = 0.21 and *Φ*_*ij*2_ = 0.26; White Mtns., code HM), and three sibling pairs for *B. huntii* across three different sites, two occurring in 2018 (*Φ*_*ij*_ = 0.22, 0.32; Magdalena and Sandia Mtns., codes MM and SC) and one in 2021 (*Φ*_*ij*_ = 0.24; White Mtns., site code HM).

### (b) Genetic diversity and inbreeding

*Bombus centralis* exhibited a strongly significant population structure (*F_ST_* = 0.33, *p* < 0.001), while *B. huntii* exhibited weak, but significant, population structure (*F_ST_* = 0.04, *p* < 0.001) (figure 3; supplementary material, table S3). The *B. centralis* population sampled at the southwestern-most site in the Pinaleño Mountains (code PL) was the most highly differentiated (pairwise *F_ST_* > 0.4 across site comparisons; figures 3a and 4a; supplementary material, table S5), though high differentiation was similarly found for populations sampled in the northeast Sandias and Pecos Mountains (codes SC and JC) (figures 3a and 4a). The other three, more central *B. centralis* populations in the Magdalena, San Mateo and White Mountains (codes MM, SM and HM) were less differentiated and had higher levels of ancestry shared; however, these populations were still distinct from one another with 100% branch support (figure 4a; supplementary material, figure S2a). Private alleles were detected in 100% of the *B. centralis* populations, with a mean of 77.12 private alleles per individual (figure 3c; supplementary material, table S6). Two *B. huntii* populations from nearby meadows in the Sacramento Mountains (codes SS, UK) shared particularly high levels of coancestry with one another, and some structuring was present within other *B. huntii* populations from the White, San Mateo and Sandias Mountains (codes HM, SM and SC) (figures 3b and 4b). We detected private alleles in only 33% of the populations, with a mean of 3.92 private alleles per individual (codes HM and SC) (figure 3d; supplementary material, table S6).

**Figure 2.**
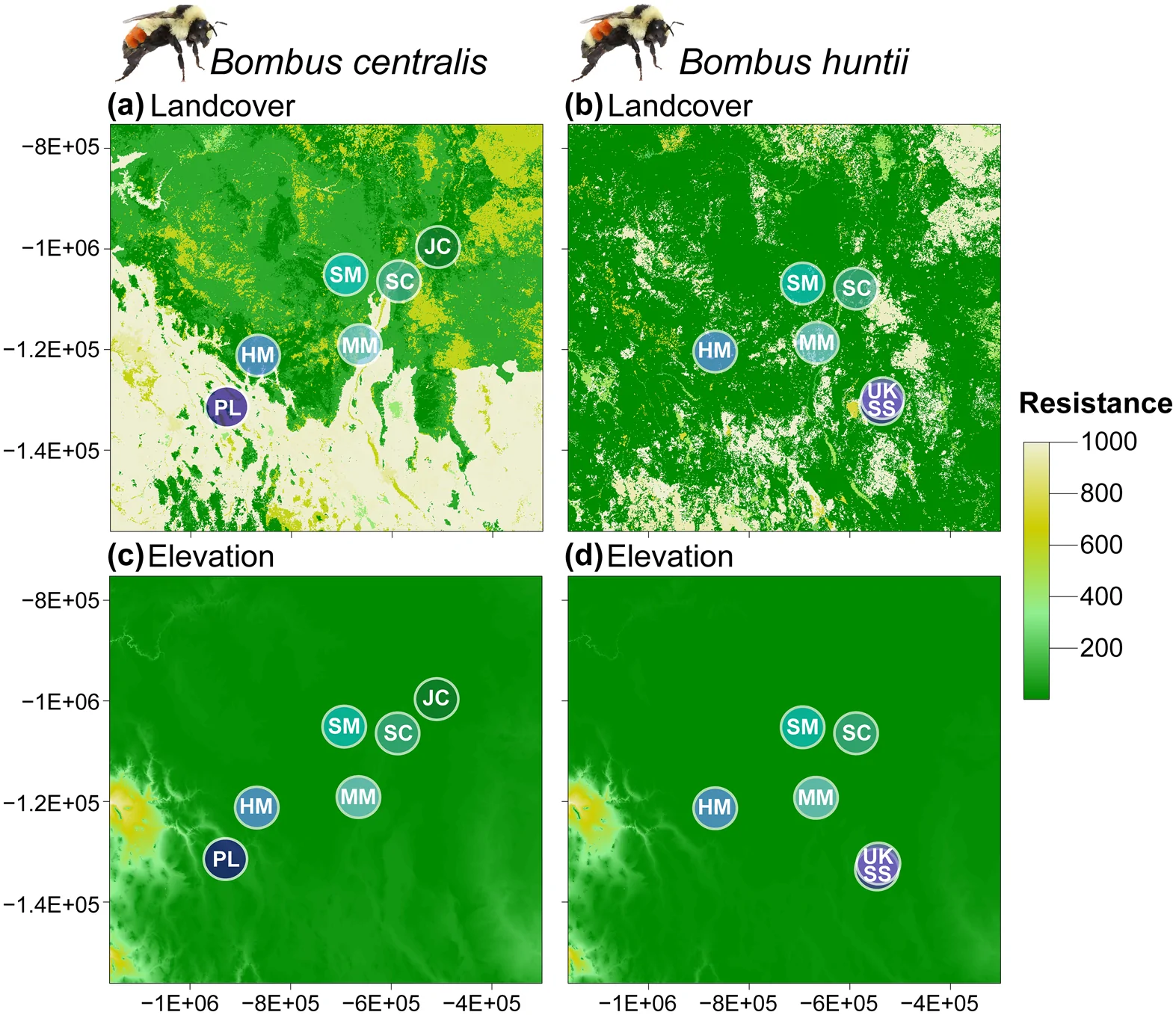
Land cover and elevation resistance layers generated from resistanceGA analyses for *B. centralis* (a, c) and *B. huntii* (b, d). Assigned resistance values are indicated in a gradient from dark green to light yellow, where darker shades represent low resistance and lighter shades represent high resistance to gene flow.

**Figure 3.**
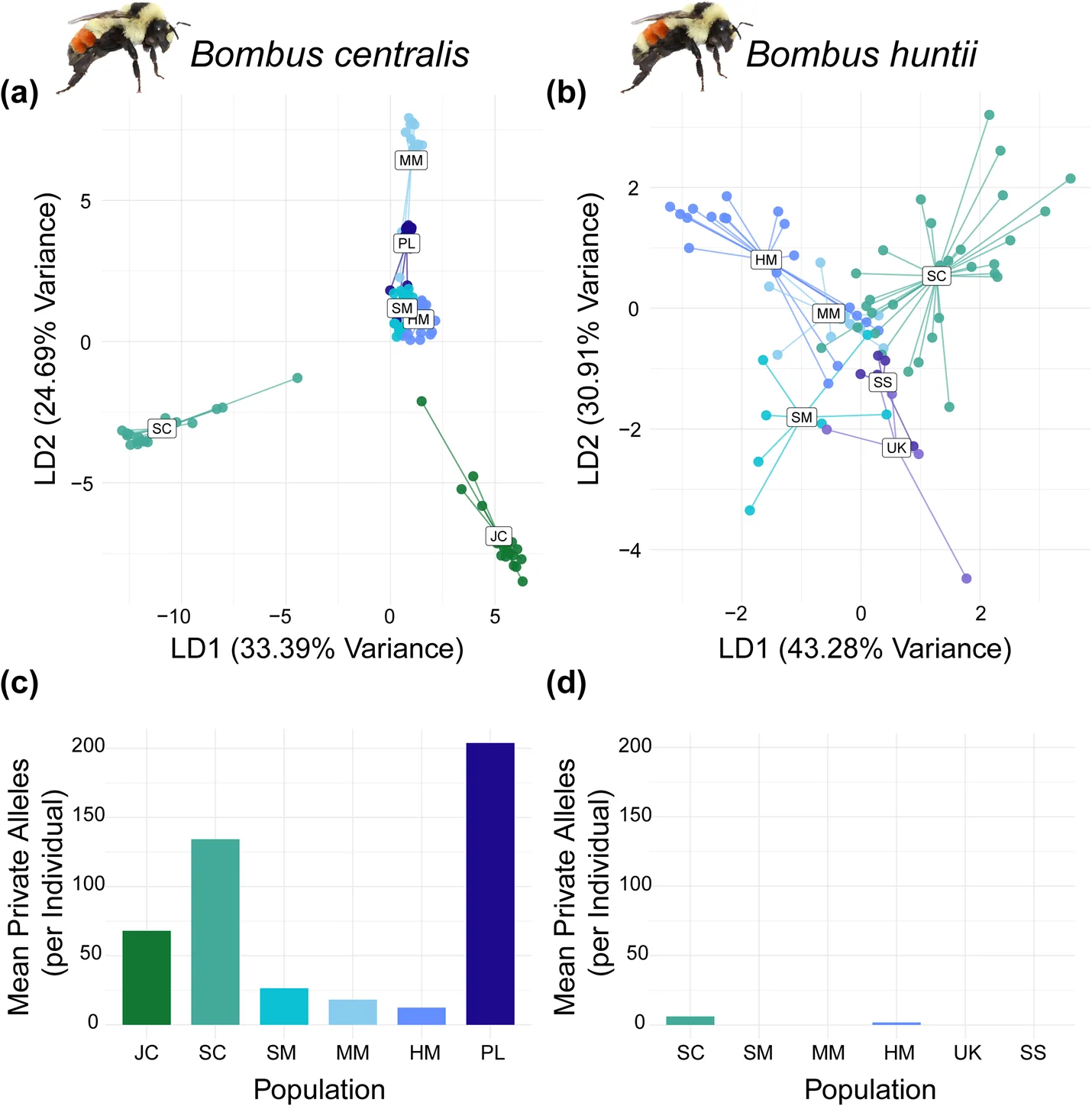
Scatter plot of DAPC showing genetic clustering for (a) *B. centralis* and (b) *B. huntii*. Axes represent the first (x-axis) and second (y-axis) loadings with the per cent of among-population variance explained by the loading indicated. Points represent individual genotypes and are connected to their population centroid. Note the differences in scale between (a) and (b). Mean number of private alleles for (c) *B. centralis* and (d) *B. huntii* populations normalized by the number of individuals in that population.

**Figure 4.**
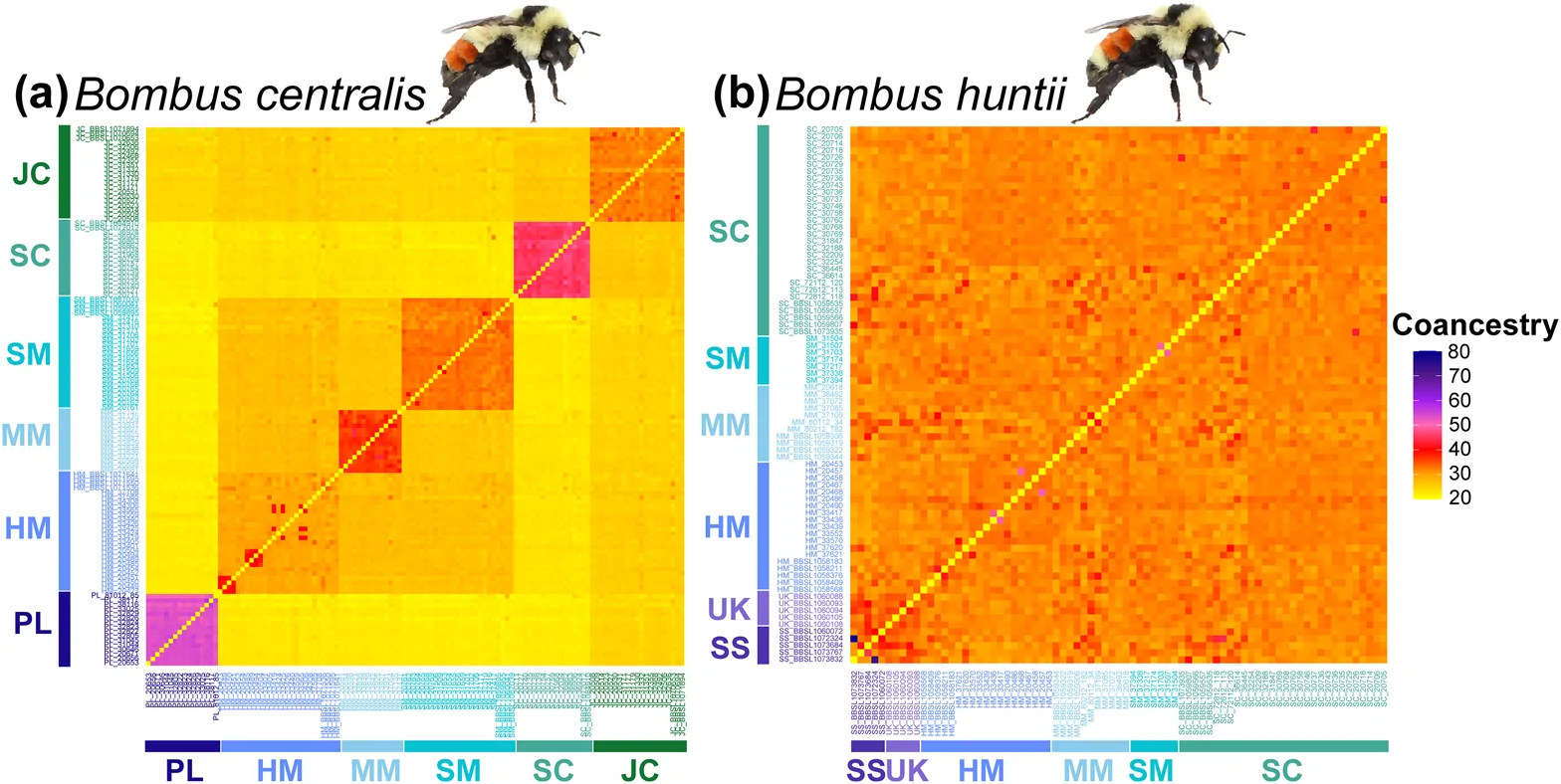
Clustered fineRADstructure coancestry matrix for (a) *B. centralis* and (b) *B. huntii*. Each box represents the coancestry coefficient between individuals. Coefficients are colour coded to designate low (yellow) to high (blue) levels of recent shared ancestry. Population assignment of individuals is indicated by colour and site code on both axes.

Inbreeding levels were high and significant for both species, but lower for *B. centralis* (*F_IS_* = 0.16, *p* < 0.001) compared with *B. huntii* (*F_IS_* = 0.39, *p* < 0.001). *Bombus centralis* exhibited reduced within-population expected diversity (*H_S_* = 0.19) and observed genotypic frequencies (*H_O_* = 0.16), demonstrating that within-population gene diversity represented only a portion of the total expected diversity (*H_T_* = 0.28) (supplementary material, table S3). By contrast, *B. huntii* populations were expected to represent nearly the total gene diversity (*H_S_* = 0.27, *H_T_* = 0.28); however, this allelic diversity was not reflected in the observed genotypic frequencies (*H_O_* = 0.16), further supporting signatures of non-random mating.

### (c) Isolation by distance or resistance

Resistance by both land cover and elevation correlated with pairwise *F_ST_* for both *B. centralis* (land cover: est. = 0.0023, *p* = 0.0033; elevation: est. = 0.036, *p* = 0.0091) and *B. huntii* (land cover: est. = 0.036, *p* < 0.001; elevation: est. = 0.044, *p* < 0.001) (figure 2; supplementary material, table S8). By contrast, Euclidean distance predicted pairwise *F_ST_* only for *B. huntii* (est. = 0.017, *p* = 0.0059). The best supported model for both species was the land cover resistance model (*B. centralis* land cover: AICc = –30.349 and *B. huntii* land cover: AICc = –69.856), followed by elevation resistance, then geographic distance (supplementary material, table S8). For *B. centralis*, temperate needle leaf forest, temperate deciduous forest, mixed forest and temperate shrubland were classified by resistanceGA as low resistance (resistance less than 333), while tropical shrubland, tropical grassland and urban land cover provided the highest resistance (resistance greater than 666) (supplementary material, table S9); all other land cover types were classified as intermediate resistance (resistance 333–666). By contrast, *B. huntii* showed low resistance to temperate needle leaf forest, all shrubland classes, wetland, water and urban land cover (resistance less than 333), and high resistance to nearly all other classes (resistance greater than 666), barring intermediate resistance to mixed forest and cropland (supplementary material, table S9).

## 4. Discussion

In this study, we leveraged an iconic sky island model system to compare genomic structure across two highly similar bumble bee species that differ only in their elevational associations, and we found drastically divergent patterns of structure and predicted drivers of differentiation. Specifically, we found that *B. centralis* populations were highly structured, all contained private alleles, and that only IBR, not IBD, explained differentiation between populations. By contrast, *B. huntii* exhibited low genomic structure, few private alleles, but both IBR and IBD explained the little differentiation detected. Across both species, we found that land cover resistance was the most predictive factor for differentiation, and we also derived ecologically distinct resistance assignments that corroborated literature-based habitat associations [50,52]. Evidence of inbreeding was detected in most populations for both species, though levels were consistently lower for *B. centralis*. Overall, our genomic structure and resistance analyses reveal the important role of ecological niche differentiation as a driver of distinct demographic and dispersal processes for co-occurring, climate-sensitive species.

First, despite similarities in size and life history, we observe vastly different overall patterns of genomic structuring between *B. centralis* and *B. huntii*. Like most other temperate bumble bees, *B. huntii* had little structure [11,19,29,30,78,79] and few, if any, private alleles within populations. This low differentiation aligns with another study in *B. huntii* by Koch and colleagues [34], which found that populations within the same geographic region as our study engaged in substantial gene flow. In our study, in contrast to *B. huntii*, we found high differentiation between all *B. centralis* populations, and we documented private alleles in all populations. While past research has not compared multiple bumble bee species across the sky islands, previous comparative bee studies have revealed that slight variation in ecological niche space can result in vastly different overarching genomic structures [11,66] and patterns of selection [80], with one study further identifying impacts on DNA methylation variation [81]. Comparative studies on other insects within sky island systems have also found differences in structure between similar species resulting from slight niche-breadth differences. For example, Halbritter and colleagues [10] found high differentiation for a host-plant specialist pine-feeding butterfly in the same sky islands system, whereas the more generalist butterfly showed evidence of panmixia. Similarly, our results support the idea that *B. centralis* is more spatially restricted to higher elevations, probably owing to its dependence on temperate habitats, suggesting that it may be more specialized than *B. huntii.* These results highlight the relevance of understanding even small differences in habitat use among species, including those broadly considered to be generalists.

Importantly, our study provides powerful evidence that land cover resistance is the primary driver of differentiation for two bumble bee species in a sky island system, whereas IBD-only models had mixed support. While it might be expected that IBD and IBR would be less predictive for *B. huntii*, which had low overall genomic structuring, Koch and colleagues [34] similarly found support for both IBD and bioclimatic-data-based IBR within one cluster of the same species despite its low structure (for bioclimatic resistance testing, see figure S4 and table S8 in the supplementary material). Indeed, our study contributes to a growing body of literature supporting both IBD and IBR driven by climate among montane bumble bees across a wide range of genomic structure [11,35,82]. However, because we find that land cover resistance is a stronger predictor of differentiation than elevation for both *B. huntii* and *B. centralis*, we suspect that differences in vegetative cover are the main source of resistance for both species in this study region. Interestingly, although both species were identified as experiencing resistance at lower elevations, their resistance to land cover classes varied widely. Most studies evaluating gene flow resistance for bumble bees assume that all natural land cover types—including forest, grassland and scrubland—offer similar, low levels of resistance to dispersal, while more extreme anthropogenic disturbance (e.g. urbanization and agricultural intensification) contributes to high resistance [19,30,31]. Our use of resistanceGA to assign resistance values without *a priori* assumptions, particularly in a system dominated by high variation in natural habitat cover, allowed for the finer scale identification of habitat types that assist or impede gene flow for bumble bees, and largely confirms the associations described for both species in the literature [50,52]. Further, this comparative study demonstrates that two species with similar physical traits and life histories, but distinct habitat-based ecological associations, indeed interact differently with the same landscapes over evolutionary time scales.

Our coancestry analyses also suggest distinct fine-scale drivers of structuring for both *B. centralis* and *B. huntii*. We detected moderate levels of coancestry across the *B. huntii* populations sampled from the Sacramento Mountains in New Mexico and some private alleles among individuals within the Sandias Mountains; similarly, the *B. centralis* population within the Sandias Mountains contained many private alleles. The Sandias are bordered by a highly urbanized area (Albuquerque, NM to the west) that was identified as causing high resistance for *B. centralis* in our study, supporting similar findings for the negative impacts of urbanization on gene flow across bee species [3,19,30,32,33], but see [72,83]. While urbanization was not found to impede *B. huntii* in our study, the east side of this range is dominated by grassland habitat that was found to create resistance for this species, suggesting that the Sandias Mountains may contribute to the isolation of populations for both species given its positioning between an urban centre and grasslands. Despite the overall high genomic structure among *B. centralis* populations, we detected recent coancestry among individuals in the White, Magdalena and San Mateo Mountains, suggesting more recent gene flow and potential dispersal corridors supported by continuous temperate habitat. In particular, the population on the Mogollon Rim in the White Mountains had the least structure among the *B. centralis* populations sampled, the least private alleles and the highest relatedness with other populations, which may suggest that the contiguous, higher elevation habitat on the rim may support dispersal for *B. centralis* in the region. By stark contrast, the Pinaleño Mountains are home to the most distinct population in our study, which contained over 200 private alleles per individual. We speculate that this high signature of isolation is attributed to the Pinaleños being both the highest and steepest mountain range in the Madrean Archipelago [84], and owing to the dominance of tropical shrubland habitat surrounding it.

The extreme genetic isolation evident in *B. centralis* also suggests that historic migration patterns may play a particularly important role in genomic structuring within this complex montane environment. Strong population differentiation has been detected in some *Bombus* species, including *B. hortorum* [6] and *B. bifarius* [11], and significant structuring at small spatial scales has been found in *B. vosnesenskii* [19,76]. However, none of these species exhibit populations as isolated as those of *B. centralis* in the sky island study system. We were unable to assess historical migration rates in this study owing to limitations in SNP data, but we believe that repeated glaciation events during the Pleistocene allowed *B. centralis* populations to colonize the more isolated mountain ranges as refugia moved down in elevation, with Holocene warming then increasing population divergence as they moved to higher elevations, as seen across other sky island taxa [85]. Given the high differentiation we observe among *B. centralis* populations, we suspect that there has been limited to no gene flow among populations for many generations owing to high land cover resistance, with structuring historically driven by migration originating from the Rockies.

In addition to these signatures of historic gene flow, the moderate to high levels of inbreeding we detected in many populations of both *B. centralis* and *B. huntii* suggest that contemporary reproductive processes are largely within-population, and potentially with closely related individuals, as seen in other isolated bumble bee populations [29,76,86–88]. Indeed, some studies find evidence of high inbreeding levels comparable to our findings in specific, isolated populations [6,29], and Kent and colleagues [79] document high inbreeding in 44% of *B. terricola* individuals. While we find significant inbreeding in all *B. huntii* populations, three of the six *B. centralis* populations sampled in our study showed no or low inbreeding (*F_IS_* < 0.1), despite evidence of isolation. Further, across sites where both species were sampled, *B. huntii* consistently showed higher inbreeding levels. A similar pattern to *B. huntii* has been documented in the bumble bee *B. vosnesenskii* sampled in the western USA, which has high levels of male diploidy—an indicator of inbreeding—despite low regional genomic structure [76]. In their study, Schenau & Jha [76] documented high genomic structure within areas of 10 km or less and found that male diploidy was highest in areas with poor nesting habitat, suggesting localized habitat-specific breeding patterns. We suspect that, as in *B. vosnesenskii*, differences in inbreeding at the population level between our two species may indicate differences in site fidelity, though the drivers of this remain unknown. We suggest that future studies in the sky island system conduct extensive within-population sampling to understand the relationship between colony density and habitat quality. Indeed, patchiness of suitable nesting and forage habitat in other sky island systems has been associated with high levels of inbreeding [40], or a wide range of inbreeding levels [41,42,48], in species demonstrating both high or low genomic structure. Notably, Ceresa and colleagues [47] found low genomic structure, yet high inbreeding, among populations of the highly mobile white-winged snowfinch, similar to *B. huntii,* and concluded this pattern to be consistent with philopatric behaviour shared among most individuals but with few long-range dispersers in the population. While uncommonly documented, evidence of extensive dispersal over a short time has been described in two range-expanding *Bombus* species, *B. ruderatus* and *B. terrestris* [22], demonstrating the capacity for some bumble bee individuals to travel far distances. We speculate that sky island systems exhibit patchy habitats at small spatial scales that promote strong philopatric behaviour in highly mobile species, ultimately resulting in high local inbreeding and structure, even if a small subset of long-range dispersers contributes to gene flow at broader scales.

Overall, our study highlights that high-elevation species are vulnerable to isolation in sky island systems, even with high genetic connectivity at the regional scale. The Cordilleran and Madrean sky islands, like all montane systems, are especially prone to rapid habitat loss and fragmentation owing to climate change and extreme weather events, particularly fire in this region [89], which can result in further isolation for populations restricted to shrinking habitat patches [90,91]. Yanahan & Moore [92] have projected significant deforestation at high elevations related to increasing temperatures as early as 2050 in the Madrean Archipelago, as well as significant isolation for populations of five montane species spanning plants, mammals and insects, owing to habitat shifts at higher elevations. This is especially alarming for *B. centralis,* which is already experiencing extreme isolation in this region, given its restriction to higher elevation, temperate refugia. The high levels of inbreeding we documented for *B. huntii* populations suggest a high risk for inbreeding depression in this species, especially if their current genetic connectivity in the region cannot be maintained in the face of climate change. The contrasting patterns we find in regional genomic structure between these two species highlight the importance of considering even small differences in ecological niche when assessing gene flow and risk, rather than relying on broader functional classes or closely related representatives for conservation planning. Given our finding that land cover is the strongest driver of gene flow resistance in this system, and is distinct between similar co-occurring species, we suggest that future studies of montane bees closely consider land cover variation in addition to elevation when evaluating patterns of differentiation and inbreeding depression risk.

## Supplementary Material



## Data Availability

All data and code are published in the Zenodo repository [93]. Supplementary material is available online [94].
