# Supplementary material for: Comparative population genomics reveal stark differences in the genomic structure and drivers of isolation for two sky island bumble bees
Source: Proc Biol Sci. 2026 Oct 7;293(2080):20261217. doi: 10.1098/rspb.2026.1217 (PMC13641907; doi:10.1098/rspb.2026.1217)
Supplement: Supplementary file 1 [file rspb.2026.1217.zip › rspb20261217_si_004.docx]

# Supplementary Materials

# Paper title: Comparative population genomics reveal stark differences in the genomic structure and drivers of isolation for two sky island bumble bees

# Authors: Laura J. Jones, Lauren C. Ponisio, Shalene Jha

# Supplemental Methods

**NextRAD library preparation and sequencing by SNPsaurus**: Nextera flex reagent (Illumina, San Diego, CA, USA) was used to fragment the DNA and ligate the short adapter sequences to the ends of each fragment. DNA fragments were amplified for 26 cycles with extension at 73 °C using two primers, one of which matches the adapter and extends 9 sequences into the genomic DNA with a selective sequence (GTGTAGAGG) at the 3’ end. Only fragments that hybridized with the selective sequence of the primer were amplified by PCR. All nextRAD libraries were sequenced on a Nextseq2000 with one P3 lane of 122 bp reads (University of Oregon, USA). We trimmed reads using bbduk with the parameters suggested by SNPsaurus.

**Supplemental Figures**

Figure S1. Discriminant Analysis of Principal Components (DAPC) compoplots demonstrating individual ancestry proportions across inferred genetic clusters (K = 2-7) for (A) *Bombus centralis* and (B) *B.huntii*. Each vertical bar represents an individual’s membership probability to each cluster. Individuals are grouped by population. Bayesian Information Criterion (BIC) provided to compare goodness of fit.

Figure S2. Coancestry-based tree inferred using fineRADstructure for (A) *Bombus centralis* and (B) *Bombus huntii*. Tree topology reflects patterns of recent shared ancestry. Posterior population assignment probabilities are indicated.

Figure S3. Input rasters for (A) land cover, (B) elevation, (C) temperature, and (D) precipitation for resistanceGA analyses. All rasters are in World Geodetic System 1984 projection. (A) Land cover data were collected for the year 2020 at 30 m resolution from the North American Land Change Monitoring System (NALCMS) and aggregated to 300 m resolution. The land cover raster is categorical, including the following categories with associated NALCMS number assignment: 1) temperate needleleaf forest, 3) tropical evergreen forest, 4) tropical deciduous forest, 5) temperate deciduous forest, 6) mixed forest, 7) tropical shrub, 8) temperate shrub, 9) tropical grassland, 10) temperate grassland, 14) wetland, 15) cropland, 16) barren, 17) urban, and18) water. Taiga needleleaf forest (2) and all moss categories (11-13) were not present within our land cover raster extent. (B) Elevation data (meters) were collected for the year 2022 at 30 m resolution from the National Elevation Dataset (U.S. Geological Survey, EROS Data Center) and aggregated to 300 m resolution. (C) Temperature and (D) precipitation data represent the 30-year (1990-2019) normal annual average temperature (Celsius) and 30-year (1990-2019) normal annual average precipitation (millimeters), respectively, collected at 1 km resolution from DAYMET (U.S. Geological Survey, doi: 10.5066/P9E0JZ82).

Figure S4. Temperature and precipitation resistance layers generated from resistanceGA analyses for *Bombus centralis* (A, C) and *B. huntii* (B, D). Assigned resistance values are indicated in a gradient from dark green to light yellow, where darker shades represent low resistance and lighter shades represent high resistance to gene flow.

**Supplemental Tables**

Table S1. Individual metadata indicating collection information and extracted tissue. Inclusion in the final dataset after filtering and kinship removal is indicated. Asterisk (*) denotes individuals that were removed after relatedness analyses due to high kinship with other individuals.

| **Sample ID** | **Species** | **Year** | **Capture** | **Site** | **State** | **Mtn. Range** | **Meadow** | **Lat.** | **Lon.** | **Elev. (m)** | **Tissue** | **Final** |
| --- | --- | --- | --- | --- | --- | --- | --- | --- | --- | --- | --- | --- |
| PL_081012_85 | *Bombus centralis* | 2012 | Net | PL | AZ | Pinaleño | Hospital Flat Campground | 32.669 | -109.878 | 2703 | Abdomen | Yes |
| PL_080912_135 | *Bombus centralis* | 2012 | Net | PL | AZ | Pinaleño | Hospital Flat Campground | 32.669 | -109.878 | 2703 | Abdomen | No |
| PL_080912_136 | *Bombus centralis* | 2012 | Net | PL | AZ | Pinaleño | Hospital Flat Campground | 32.669 | -109.878 | 2703 | Abdomen | No |
| PL_080912_335 | *Bombus centralis* | 2012 | Net | PL | AZ | Pinaleño | Hospital Flat Campground | 32.669 | -109.878 | 2703 | Abdomen | No |
| PL_081212_151 | *Bombus centralis* | 2012 | Net | PL | AZ | Pinaleño | Hospital Flat Campground | 32.669 | -109.878 | 2703 | Abdomen | No |
| HM_BBSL1071536 | *Bombus centralis* | 2017 | Net | HM | AZ | White | Hannagan | 33.632 | -109.325 | 2793 | Abdomen | Yes |
| HM_BBSL1071550 | *Bombus centralis* | 2017 | Net | HM | AZ | White | Hannagan | 33.632 | -109.325 | 2793 | Abdomen | Yes |
| HM_BBSL1071595 | *Bombus centralis* | 2017 | Net | HM | AZ | White | Hannagan | 33.632 | -109.325 | 2793 | Abdomen | Yes |
| HM_BBSL1071641 | *Bombus centralis* | 2017 | Net | HM | AZ | White | Hannagan | 33.632 | -109.325 | 2793 | Abdomen | Yes |
| JC_BBSL1070653 | *Bombus centralis* | 2017 | Net | JC | NM | Pecos | Jack's Creek Campground | 35.855 | -105.641 | 2952 | Abdomen | Yes |
| JC_BBSL1070694 | *Bombus centralis* | 2017 | Net | JC | NM | Pecos | Jack's Creek Campground | 35.855 | -105.641 | 2952 | Abdomen | Yes |
| JC_BBSL1071894 | *Bombus centralis* | 2017 | Vane | JC | NM | Pecos | Jack's Creek Campground | 35.855 | -105.641 | 2952 | Abdomen | Yes |
| SC_BBSL1072012 | *Bombus centralis* | 2017 | Pan | SC | NM | Sandias | Kiwanis | 35.206 | -106.443 | 3091 | Abdomen | Yes |
| SC_BBSL1084910 | *Bombus centralis* | 2017 | Vane | SC | NM | Sandias | Kiwanis | 35.206 | -106.443 | 3091 | Abdomen | Yes |
| SM_BBSL1059895 | *Bombus centralis* | 2017 | Net | SM | NM | San Mateo | La Mosca Lookout Tower | 35.248 | -107.599 | 3063 | Abdomen | Yes |
| SM_BBSL1059955 | *Bombus centralis* | 2017 | Net | SM | NM | San Mateo | La Mosca Lookout Tower | 35.248 | -107.599 | 3063 | Abdomen | Yes |
| SM_BBSL1060041 | *Bombus centralis* | 2017 | Net | SM | NM | San Mateo | La Mosca Lookout Tower | 35.248 | -107.599 | 3063 | Abdomen | Yes |
| SM_BBSL1087039 | *Bombus centralis* | 2017 | Pan | SM | NM | San Mateo | La Mosca Lookout Tower | 35.248 | -107.599 | 3063 | Abdomen | Yes |
| HM_BBSL1071537 | *Bombus centralis* | 2017 | Net | HM | AZ | White | Hannagan | 33.632 | -109.325 | 2793 | Abdomen | No |
| JC_BBSL1070604 | *Bombus centralis* | 2017 | Net | JC | NM | Pecos | Jack's Creek Campground | 35.855 | -105.641 | 2952 | Abdomen | No |
| JC_BBSL1070636 | *Bombus centralis* | 2017 | Net | JC | NM | Pecos | Jack's Creek Campground | 35.855 | -105.641 | 2952 | Abdomen | No |
| MM_BBSL1059115 | *Bombus centralis* | 2017 | Net | MM | NM | Magdalena | South Baldy | 33.991 | -107.184 | 2979 | Abdomen | No |
| MM_BBSL1070264 | *Bombus centralis* | 2017 | Net | MM | NM | Magdalena | South Baldy | 33.991 | -107.184 | 2979 | Abdomen | No |
| MM_BBSL1072888 | *Bombus centralis* | 2017 | Net | MM | NM | Magdalena | South Baldy | 33.991 | -107.184 | 2979 | Abdomen | No |
| MM_BBSL1072896 | *Bombus centralis* | 2017 | Net | MM | NM | Magdalena | South Baldy | 33.991 | -107.184 | 2979 | Abdomen | No |
| MM_BBSL1074097 | *Bombus centralis* | 2017 | Net | MM | NM | Magdalena | South Baldy | 33.991 | -107.184 | 2979 | Abdomen | No |
| SC_BBSL1059555 | *Bombus centralis* | 2017 | Net | SC | NM | Sandias | Kiwanis | 35.206 | -106.443 | 3091 | Abdomen | No |
| SC_BBSL1059813 | *Bombus centralis* | 2017 | Net | SC | NM | Sandias | Kiwanis | 35.206 | -106.443 | 3091 | Abdomen | No |
| SC_BBSL1059867 | *Bombus centralis* | 2017 | Net | SC | NM | Sandias | Kiwanis | 35.206 | -106.443 | 3091 | Abdomen | No |
| SM_BBSL1059968 | *Bombus centralis* | 2017 | Net | SM | NM | San Mateo | La Mosca Lookout Tower | 35.248 | -107.599 | 3063 | Abdomen | No |
| HM_20423 | *Bombus centralis* | 2018 | Net | HM | AZ | White | Hannagan | 33.632 | -109.325 | 2793 | Gut | Yes |
| HM_20449 | *Bombus centralis* | 2018 | Net | HM | AZ | White | Hannagan | 33.632 | -109.325 | 2793 | Gut | Yes |
| HM_20450 | *Bombus centralis* | 2018 | Net | HM | AZ | White | Hannagan | 33.632 | -109.325 | 2793 | Gut | Yes |
| HM_20451 | *Bombus centralis* | 2018 | Net | HM | AZ | White | Hannagan | 33.632 | -109.325 | 2793 | Gut | Yes |
| HM_20452 | *Bombus centralis* | 2018 | Net | HM | AZ | White | Hannagan | 33.632 | -109.325 | 2793 | Gut | Yes |
| HM_20454 | *Bombus centralis* | 2018 | Net | HM | AZ | White | Hannagan | 33.632 | -109.325 | 2793 | Gut | Yes |
| HM_20485 | *Bombus centralis* | 2018 | Net | HM | AZ | White | Hannagan | 33.632 | -109.325 | 2793 | Gut | Yes |
| HM_20489 | *Bombus centralis* | 2018 | Net | HM | AZ | White | Hannagan | 33.632 | -109.325 | 2793 | Gut | Yes |
| HM_20494 | *Bombus centralis* | 2018 | Net | HM | AZ | White | Hannagan | 33.632 | -109.325 | 2793 | Gut | Yes |
| HM_20504 | *Bombus centralis* | 2018 | Net | HM | AZ | White | Hannagan | 33.632 | -109.325 | 2793 | Gut | Yes |
| JC_20508 | *Bombus centralis* | 2018 | Net | JC | NM | Pecos | Jack's Creek Campground | 35.855 | -105.641 | 2952 | Gut | Yes |
| JC_20509 | *Bombus centralis* | 2018 | Net | JC | NM | Pecos | Jack's Creek Campground | 35.855 | -105.641 | 2952 | Gut | Yes |
| JC_20514 | *Bombus centralis* | 2018 | Net | JC | NM | Pecos | Jack's Creek Campground | 35.855 | -105.641 | 2952 | Gut | Yes |
| JC_20523 | *Bombus centralis* | 2018 | Net | JC | NM | Pecos | Jack's Creek Campground | 35.855 | -105.641 | 2952 | Gut | Yes |
| JC_20527 | *Bombus centralis* | 2018 | Net | JC | NM | Pecos | Jack's Creek Campground | 35.855 | -105.641 | 2952 | Gut | Yes |
| JC_20530 | *Bombus centralis* | 2018 | Net | JC | NM | Pecos | Jack's Creek Campground | 35.855 | -105.641 | 2952 | Gut | Yes |
| JC_20531 | *Bombus centralis* | 2018 | Net | JC | NM | Pecos | Jack's Creek Campground | 35.855 | -105.641 | 2952 | Gut | Yes |
| MM_20581 | *Bombus centralis* | 2018 | Net | MM | NM | Magdalena | South Baldy | 33.991 | -107.184 | 2979 | Gut | Yes |
| MM_20594 | *Bombus centralis* | 2018 | Net | MM | NM | Magdalena | South Baldy | 33.991 | -107.184 | 2979 | Gut | Yes |
| MM_20598 | *Bombus centralis* | 2018 | Net | MM | NM | Magdalena | South Baldy | 33.991 | -107.184 | 2979 | Gut | Yes |
| PL_20653 | *Bombus centralis* | 2018 | Net | PL | AZ | Pinaleño | Hospital Flat Campground | 32.669 | -109.878 | 2703 | Gut | Yes |
| PL_20656 | *Bombus centralis* | 2018 | Net | PL | AZ | Pinaleño | Hospital Flat Campground | 32.669 | -109.878 | 2703 | Gut | Yes |
| PL_20671 | *Bombus centralis* | 2018 | Net | PL | AZ | Pinaleño | Hospital Flat Campground | 32.669 | -109.878 | 2703 | Gut | Yes |
| SC_20711 | *Bombus centralis* | 2018 | Net | SC | NM | Sandias | Kiwanis | 35.206 | -106.443 | 3091 | Gut | Yes |
| SC_20727 | *Bombus centralis* | 2018 | Net | SC | NM | Sandias | Kiwanis | 35.206 | -106.443 | 3091 | Gut | Yes |
| SC_20740 | *Bombus centralis* | 2018 | Net | SC | NM | Sandias | Kiwanis | 35.206 | -106.443 | 3091 | Gut | Yes |
| SM_20761 | *Bombus centralis* | 2018 | Net | SM | NM | San Mateo | La Mosca Lookout Tower | 35.248 | -107.599 | 3063 | Gut | Yes |
| SM_20762 | *Bombus centralis* | 2018 | Net | SM | NM | San Mateo | La Mosca Lookout Tower | 35.248 | -107.599 | 3063 | Gut | Yes |
| SM_20763 | *Bombus centralis* | 2018 | Net | SM | NM | San Mateo | La Mosca Lookout Tower | 35.248 | -107.599 | 3063 | Gut | Yes |
| SM_20764 | *Bombus centralis* | 2018 | Net | SM | NM | San Mateo | La Mosca Lookout Tower | 35.248 | -107.599 | 3063 | Gut | Yes |
| SM_20765 | *Bombus centralis* | 2018 | Net | SM | NM | San Mateo | La Mosca Lookout Tower | 35.248 | -107.599 | 3063 | Gut | Yes |
| SM_20768 | *Bombus centralis* | 2018 | Net | SM | NM | San Mateo | La Mosca Lookout Tower | 35.248 | -107.599 | 3063 | Gut | Yes |
| SM_20769 | *Bombus centralis* | 2018 | Net | SM | NM | San Mateo | La Mosca Lookout Tower | 35.248 | -107.599 | 3063 | Gut | Yes |
| HM_20502 | *Bombus centralis* | 2018 | Net | HM | AZ | White | Hannagan | 33.632 | -109.325 | 2793 | Gut | No* |
| HM_33401 | *Bombus centralis* | 2021 | Net | HM | AZ | White | Hannagan | 33.632 | -109.325 | 2793 | Gut | Yes |
| HM_33405 | *Bombus centralis* | 2021 | Net | HM | AZ | White | Hannagan | 33.632 | -109.325 | 2793 | Gut | Yes |
| HM_33419 | *Bombus centralis* | 2021 | Net | HM | AZ | White | Hannagan | 33.632 | -109.325 | 2793 | Gut | Yes |
| HM_33424 | *Bombus centralis* | 2021 | Net | HM | AZ | White | Hannagan | 33.632 | -109.325 | 2793 | Gut | Yes |
| HM_33425 | *Bombus centralis* | 2021 | Net | HM | AZ | White | Hannagan | 33.632 | -109.325 | 2793 | Gut | Yes |
| HM_33426 | *Bombus centralis* | 2021 | Net | HM | AZ | White | Hannagan | 33.632 | -109.325 | 2793 | Gut | Yes |
| HM_33440 | *Bombus centralis* | 2021 | Net | HM | AZ | White | Hannagan | 33.632 | -109.325 | 2793 | Gut | Yes |
| HM_33559 | *Bombus centralis* | 2021 | Net | HM | AZ | White | Hannagan | 33.632 | -109.325 | 2793 | Gut | Yes |
| HM_33565 | *Bombus centralis* | 2021 | Net | HM | AZ | White | Hannagan | 33.632 | -109.325 | 2793 | Gut | Yes |
| HM_34306 | *Bombus centralis* | 2021 | Net | HM | AZ | White | Hannagan | 33.632 | -109.325 | 2793 | Gut | Yes |
| HM_34308 | *Bombus centralis* | 2021 | Net | HM | AZ | White | Hannagan | 33.632 | -109.325 | 2793 | Gut | Yes |
| JC_31171 | *Bombus centralis* | 2021 | Net | JC | NM | Pecos | Jack's Creek Campground | 35.855 | -105.641 | 2952 | Gut | Yes |
| JC_31177 | *Bombus centralis* | 2021 | Net | JC | NM | Pecos | Jack's Creek Campground | 35.855 | -105.641 | 2952 | Gut | Yes |
| JC_31179 | *Bombus centralis* | 2021 | Net | JC | NM | Pecos | Jack's Creek Campground | 35.855 | -105.641 | 2952 | Gut | Yes |
| JC_31330 | *Bombus centralis* | 2021 | Net | JC | NM | Pecos | Jack's Creek Campground | 35.855 | -105.641 | 2952 | Gut | Yes |
| JC_31332 | *Bombus centralis* | 2021 | Net | JC | NM | Pecos | Jack's Creek Campground | 35.855 | -105.641 | 2952 | Gut | Yes |
| JC_31351 | *Bombus centralis* | 2021 | Net | JC | NM | Pecos | Jack's Creek Campground | 35.855 | -105.641 | 2952 | Gut | Yes |
| JC_32355 | *Bombus centralis* | 2021 | Net | JC | NM | Pecos | Jack's Creek Campground | 35.855 | -105.641 | 2952 | Gut | Yes |
| JC_32468 | *Bombus centralis* | 2021 | Net | JC | NM | Pecos | Jack's Creek Campground | 35.855 | -105.641 | 2952 | Gut | Yes |
| JC_32492 | *Bombus centralis* | 2021 | Net | JC | NM | Pecos | Jack's Creek Campground | 35.855 | -105.641 | 2952 | Gut | Yes |
| JC_32506 | *Bombus centralis* | 2021 | Net | JC | NM | Pecos | Jack's Creek Campground | 35.855 | -105.641 | 2952 | Gut | Yes |
| JC_32636 | *Bombus centralis* | 2021 | Net | JC | NM | Pecos | Jack's Creek Campground | 35.855 | -105.641 | 2952 | Gut | Yes |
| MM_33221 | *Bombus centralis* | 2021 | Net | MM | NM | Magdalena | South Baldy | 33.991 | -107.184 | 2979 | Gut | Yes |
| MM_33836 | *Bombus centralis* | 2021 | Net | MM | NM | Magdalena | South Baldy | 33.991 | -107.184 | 2979 | Gut | Yes |
| MM_33838 | *Bombus centralis* | 2021 | Net | MM | NM | Magdalena | South Baldy | 33.991 | -107.184 | 2979 | Gut | Yes |
| MM_33839 | *Bombus centralis* | 2021 | Net | MM | NM | Magdalena | South Baldy | 33.991 | -107.184 | 2979 | Gut | Yes |
| MM_33842 | *Bombus centralis* | 2021 | Net | MM | NM | Magdalena | South Baldy | 33.991 | -107.184 | 2979 | Gut | Yes |
| MM_33925 | *Bombus centralis* | 2021 | Net | MM | NM | Magdalena | South Baldy | 33.991 | -107.184 | 2979 | Gut | Yes |
| MM_33927 | *Bombus centralis* | 2021 | Net | MM | NM | Magdalena | South Baldy | 33.991 | -107.184 | 2979 | Gut | Yes |
| MM_33931 | *Bombus centralis* | 2021 | Net | MM | NM | Magdalena | South Baldy | 33.991 | -107.184 | 2979 | Gut | Yes |
| PL_30646 | *Bombus centralis* | 2021 | Net | PL | AZ | Pinaleño | Hospital Flat Campground | 32.669 | -109.878 | 2703 | Gut | Yes |
| PL_31044 | *Bombus centralis* | 2021 | Net | PL | AZ | Pinaleño | Hospital Flat Campground | 32.669 | -109.878 | 2703 | Gut | Yes |
| PL_31045 | *Bombus centralis* | 2021 | Net | PL | AZ | Pinaleño | Hospital Flat Campground | 32.669 | -109.878 | 2703 | Gut | Yes |
| PL_32805 | *Bombus centralis* | 2021 | Net | PL | AZ | Pinaleño | Hospital Flat Campground | 32.669 | -109.878 | 2703 | Gut | Yes |
| PL_32822 | *Bombus centralis* | 2021 | Net | PL | AZ | Pinaleño | Hospital Flat Campground | 32.669 | -109.878 | 2703 | Gut | Yes |
| PL_32823 | *Bombus centralis* | 2021 | Net | PL | AZ | Pinaleño | Hospital Flat Campground | 32.669 | -109.878 | 2703 | Gut | Yes |
| PL_32824 | *Bombus centralis* | 2021 | Net | PL | AZ | Pinaleño | Hospital Flat Campground | 32.669 | -109.878 | 2703 | Gut | Yes |
| PL_32826 | *Bombus centralis* | 2021 | Net | PL | AZ | Pinaleño | Hospital Flat Campground | 32.669 | -109.878 | 2703 | Gut | Yes |
| PL_32829 | *Bombus centralis* | 2021 | Net | PL | AZ | Pinaleño | Hospital Flat Campground | 32.669 | -109.878 | 2703 | Gut | Yes |
| PL_33025 | *Bombus centralis* | 2021 | Net | PL | AZ | Pinaleño | Hospital Flat Campground | 32.669 | -109.878 | 2703 | Gut | Yes |
| SC_30735 | *Bombus centralis* | 2021 | Net | SC | NM | Sandias | Kiwanis | 35.206 | -106.443 | 3091 | Gut | Yes |
| SC_30738 | *Bombus centralis* | 2021 | Net | SC | NM | Sandias | Kiwanis | 35.206 | -106.443 | 3091 | Gut | Yes |
| SC_30739 | *Bombus centralis* | 2021 | Net | SC | NM | Sandias | Kiwanis | 35.206 | -106.443 | 3091 | Gut | Yes |
| SC_30754 | *Bombus centralis* | 2021 | Net | SC | NM | Sandias | Kiwanis | 35.206 | -106.443 | 3091 | Gut | Yes |
| SC_30757 | *Bombus centralis* | 2021 | Net | SC | NM | Sandias | Kiwanis | 35.206 | -106.443 | 3091 | Gut | Yes |
| SC_30759 | *Bombus centralis* | 2021 | Net | SC | NM | Sandias | Kiwanis | 35.206 | -106.443 | 3091 | Gut | Yes |
| SC_31968 | *Bombus centralis* | 2021 | Net | SC | NM | Sandias | Kiwanis | 35.206 | -106.443 | 3091 | Gut | Yes |
| SC_32034 | *Bombus centralis* | 2021 | Net | SC | NM | Sandias | Kiwanis | 35.206 | -106.443 | 3091 | Gut | Yes |
| SM_31506 | *Bombus centralis* | 2021 | Net | SM | NM | San Mateo | La Mosca Lookout Tower | 35.248 | -107.599 | 3063 | Gut | Yes |
| SM_31509 | *Bombus centralis* | 2021 | Net | SM | NM | San Mateo | La Mosca Lookout Tower | 35.248 | -107.599 | 3063 | Gut | Yes |
| SM_31653 | *Bombus centralis* | 2021 | Net | SM | NM | San Mateo | La Mosca Lookout Tower | 35.248 | -107.599 | 3063 | Gut | Yes |
| SM_31654 | *Bombus centralis* | 2021 | Net | SM | NM | San Mateo | La Mosca Lookout Tower | 35.248 | -107.599 | 3063 | Gut | Yes |
| SM_31655 | *Bombus centralis* | 2021 | Net | SM | NM | San Mateo | La Mosca Lookout Tower | 35.248 | -107.599 | 3063 | Gut | Yes |
| SM_31656 | *Bombus centralis* | 2021 | Net | SM | NM | San Mateo | La Mosca Lookout Tower | 35.248 | -107.599 | 3063 | Gut | Yes |
| SM_31659 | *Bombus centralis* | 2021 | Net | SM | NM | San Mateo | La Mosca Lookout Tower | 35.248 | -107.599 | 3063 | Gut | Yes |
| SM_31701 | *Bombus centralis* | 2021 | Net | SM | NM | San Mateo | La Mosca Lookout Tower | 35.248 | -107.599 | 3063 | Gut | Yes |
| SM_31702 | *Bombus centralis* | 2021 | Net | SM | NM | San Mateo | La Mosca Lookout Tower | 35.248 | -107.599 | 3063 | Gut | Yes |
| SM_31706 | *Bombus centralis* | 2021 | Net | SM | NM | San Mateo | La Mosca Lookout Tower | 35.248 | -107.599 | 3063 | Gut | Yes |
| PL_32827 | *Bombus centralis* | 2021 | Net | PL | AZ | Pinaleño | Hospital Flat Campground | 32.669 | -109.878 | 2703 | Gut | No |
| SC_30733 | *Bombus centralis* | 2021 | Net | SC | NM | Sandias | Kiwanis | 35.206 | -106.443 | 3091 | Gut | No |
| SC_30741 | *Bombus centralis* | 2021 | Net | SC | NM | Sandias | Kiwanis | 35.206 | -106.443 | 3091 | Gut | No |
| SC_30767 | *Bombus centralis* | 2021 | Net | SC | NM | Sandias | Kiwanis | 35.206 | -106.443 | 3091 | Gut | No |
| HM_37585 | *Bombus centralis* | 2022 | Net | HM | AZ | White | Hannagan | 33.632 | -109.325 | 2793 | Abdomen | Yes |
| HM_37708 | *Bombus centralis* | 2022 | Net | HM | AZ | White | Hannagan | 33.632 | -109.325 | 2793 | Abdomen | Yes |
| MM_37054 | *Bombus centralis* | 2022 | Net | MM | NM | Magdalena | South Baldy | 33.991 | -107.184 | 2979 | Abdomen | Yes |
| MM_37135 | *Bombus centralis* | 2022 | Net | MM | NM | Magdalena | South Baldy | 33.991 | -107.184 | 2979 | Abdomen | Yes |
| MM_37137 | *Bombus centralis* | 2022 | Net | MM | NM | Magdalena | South Baldy | 33.991 | -107.184 | 2979 | Abdomen | Yes |
| PL_38116 | *Bombus centralis* | 2022 | Net | PL | AZ | Pinaleño | Hospital Flat Campground | 32.669 | -109.878 | 2703 | Abdomen | Yes |
| PL_38117 | *Bombus centralis* | 2022 | Net | PL | AZ | Pinaleño | Hospital Flat Campground | 32.669 | -109.878 | 2703 | Abdomen | Yes |
| SC_36862 | *Bombus centralis* | 2022 | Net | SC | NM | Sandias | Kiwanis | 35.206 | -106.443 | 3091 | Abdomen | Yes |
| SC_36863 | *Bombus centralis* | 2022 | Net | SC | NM | Sandias | Kiwanis | 35.206 | -106.443 | 3091 | Abdomen | Yes |
| SC_36906 | *Bombus centralis* | 2022 | Net | SC | NM | Sandias | Kiwanis | 35.206 | -106.443 | 3091 | Abdomen | Yes |
| SC_36924 | *Bombus centralis* | 2022 | Net | SC | NM | Sandias | Kiwanis | 35.206 | -106.443 | 3091 | Abdomen | Yes |
| SM_37171 | *Bombus centralis* | 2022 | Net | SM | NM | San Mateo | La Mosca Lookout Tower | 35.248 | -107.599 | 3063 | Abdomen | Yes |
| SM_37310 | *Bombus centralis* | 2022 | Net | SM | NM | San Mateo | La Mosca Lookout Tower | 35.248 | -107.599 | 3063 | Abdomen | Yes |
| SM_37416 | *Bombus centralis* | 2022 | Net | SM | NM | San Mateo | La Mosca Lookout Tower | 35.248 | -107.599 | 3063 | Abdomen | Yes |
| SM_37417 | *Bombus centralis* | 2022 | Net | SM | NM | San Mateo | La Mosca Lookout Tower | 35.248 | -107.599 | 3063 | Abdomen | Yes |
| HM_37642 | *Bombus centralis* | 2022 | Net | HM | AZ | White | Hannagan | 33.632 | -109.325 | 2793 | Abdomen | No |
| HM_37691 | *Bombus centralis* | 2022 | Net | HM | AZ | White | Hannagan | 33.632 | -109.325 | 2793 | Abdomen | No |
| HM_37917 | *Bombus centralis* | 2022 | Net | HM | AZ | White | Hannagan | 33.632 | -109.325 | 2793 | Abdomen | No |
| MM_37045 | *Bombus centralis* | 2022 | Net | MM | NM | Magdalena | South Baldy | 33.991 | -107.184 | 2979 | Abdomen | No |
| MM_37048 | *Bombus centralis* | 2022 | Net | MM | NM | Magdalena | South Baldy | 33.991 | -107.184 | 2979 | Abdomen | No |
| PL_38102 | *Bombus centralis* | 2022 | Net | PL | AZ | Pinaleño | Hospital Flat Campground | 32.669 | -109.878 | 2703 | Abdomen | No |
| PL_38104 | *Bombus centralis* | 2022 | Net | PL | AZ | Pinaleño | Hospital Flat Campground | 32.669 | -109.878 | 2703 | Abdomen | No |
| PL_38110 | *Bombus centralis* | 2022 | Net | PL | AZ | Pinaleño | Hospital Flat Campground | 32.669 | -109.878 | 2703 | Abdomen | No |
| SC_36872 | *Bombus centralis* | 2022 | Net | SC | NM | Sandias | Kiwanis | 35.206 | -106.443 | 3091 | Abdomen | No |
| SM_37176 | *Bombus centralis* | 2022 | Net | SM | NM | San Mateo | La Mosca Lookout Tower | 35.248 | -107.599 | 3063 | Abdomen | No |
| MM_080112_34 | *Bombus huntii* | 2012 | Net | MM | NM | Magdalena | South Baldy | 33.991 | -107.184 | 2979 | Abdomen | Yes |
| MM_080212_182 | *Bombus huntii* | 2012 | Net | MM | NM | Magdalena | South Baldy | 33.991 | -107.184 | 2979 | Abdomen | Yes |
| SC_072112_120 | *Bombus huntii* | 2012 | Net | SC | NM | Sandias | Kiwanis | 35.206 | -106.443 | 3091 | Abdomen | Yes |
| SC_072612_113 | *Bombus huntii* | 2012 | Net | SC | NM | Sandias | Kiwanis | 35.206 | -106.443 | 3091 | Abdomen | Yes |
| SC_072812_118 | *Bombus huntii* | 2012 | Net | SC | NM | Sandias | Kiwanis | 35.206 | -106.443 | 3091 | Abdomen | Yes |
| MM_080112_93 | *Bombus huntii* | 2012 | Net | MM | NM | Magdalena | South Baldy | 33.991 | -107.184 | 2979 | Abdomen | No |
| MM_080112_95 | *Bombus huntii* | 2012 | Net | MM | NM | Magdalena | South Baldy | 33.991 | -107.184 | 2979 | Abdomen | No |
| MM_080212_116 | *Bombus huntii* | 2012 | Net | MM | NM | Magdalena | South Baldy | 33.991 | -107.184 | 2979 | Abdomen | No |
| SC_072112_53 | *Bombus huntii* | 2012 | Net | SC | NM | Sandias | Kiwanis | 35.206 | -106.443 | 3091 | Abdomen | No |
| SC_072612_174 | *Bombus huntii* | 2012 | Net | SC | NM | Sandias | Kiwanis | 35.206 | -106.443 | 3091 | Abdomen | No |
| HM_BBSL1058183 | *Bombus huntii* | 2017 | Net | HM | AZ | White | Hannagan | 33.632 | -109.325 | 2793 | Abdomen | Yes |
| HM_BBSL1058211 | *Bombus huntii* | 2017 | Net | HM | AZ | White | Hannagan | 33.632 | -109.325 | 2793 | Abdomen | Yes |
| HM_BBSL1058376 | *Bombus huntii* | 2017 | Net | HM | AZ | White | Hannagan | 33.632 | -109.325 | 2793 | Abdomen | Yes |
| HM_BBSL1058409 | *Bombus huntii* | 2017 | Net | HM | AZ | White | Hannagan | 33.632 | -109.325 | 2793 | Abdomen | Yes |
| HM_BBSL1058568 | *Bombus huntii* | 2017 | Net | HM | AZ | White | Hannagan | 33.632 | -109.325 | 2793 | Abdomen | Yes |
| MM_BBSL1059306 | *Bombus huntii* | 2017 | Net | MM | NM | Magdalena | South Baldy | 33.991 | -107.184 | 2979 | Abdomen | Yes |
| MM_BBSL1059319 | *Bombus huntii* | 2017 | Net | MM | NM | Magdalena | South Baldy | 33.991 | -107.184 | 2979 | Abdomen | Yes |
| MM_BBSL1059322 | *Bombus huntii* | 2017 | Net | MM | NM | Magdalena | South Baldy | 33.991 | -107.184 | 2979 | Abdomen | Yes |
| MM_BBSL1059344 | *Bombus huntii* | 2017 | Net | MM | NM | Magdalena | South Baldy | 33.991 | -107.184 | 2979 | Abdomen | Yes |
| SC_BBSL1059535 | *Bombus huntii* | 2017 | Net | SC | NM | Sandias | Kiwanis | 35.206 | -106.443 | 3091 | Abdomen | Yes |
| SC_BBSL1059557 | *Bombus huntii* | 2017 | Net | SC | NM | Sandias | Kiwanis | 35.206 | -106.443 | 3091 | Abdomen | Yes |
| SC_BBSL1059566 | *Bombus huntii* | 2017 | Net | SC | NM | Sandias | Kiwanis | 35.206 | -106.443 | 3091 | Abdomen | Yes |
| SC_BBSL1059807 | *Bombus huntii* | 2017 | Net | SC | NM | Sandias | Kiwanis | 35.206 | -106.443 | 3091 | Abdomen | Yes |
| SC_BBSL1073935 | *Bombus huntii* | 2017 | Net | SC | NM | Sandias | Kiwanis | 35.206 | -106.443 | 3091 | Abdomen | Yes |
| SS_BBSL1060072 | *Bombus huntii* | 2017 | Net | SS | NM | Sacramento | Sunspot | 32.789 | -105.786 | 2865 | Abdomen | Yes |
| SS_BBSL1072324 | *Bombus huntii* | 2017 | Vane | SS | NM | Sacramento | Sunspot | 32.804 | -105.804 | 2897 | Abdomen | Yes |
| SS_BBSL1073684 | *Bombus huntii* | 2017 | Vane | SS | NM | Sacramento | Sunspot | 32.804 | -105.804 | 2897 | Abdomen | Yes |
| SS_BBSL1073767 | *Bombus huntii* | 2017 | Vane | SS | NM | Sacramento | Sunspot | 32.804 | -105.804 | 2897 | Abdomen | Yes |
| SS_BBSL1073832 | *Bombus huntii* | 2017 | Vane | SS | NM | Sacramento | Sunspot | 32.804 | -105.804 | 2897 | Abdomen | Yes |
| UK_BBSL1060088 | *Bombus huntii* | 2017 | Net | UK | NM | Sacramento | Upper Karr Campground | 32.882 | -105.775 | 2760 | Abdomen | Yes |
| UK_BBSL1060093 | *Bombus huntii* | 2017 | Net | UK | NM | Sacramento | Upper Karr Campground | 32.882 | -105.775 | 2760 | Abdomen | Yes |
| UK_BBSL1060094 | *Bombus huntii* | 2017 | Net | UK | NM | Sacramento | Upper Karr Campground | 32.882 | -105.775 | 2760 | Abdomen | Yes |
| UK_BBSL1060105 | *Bombus huntii* | 2017 | Net | UK | NM | Sacramento | Upper Karr Campground | 32.882 | -105.775 | 2760 | Abdomen | Yes |
| UK_BBSL1060108 | *Bombus huntii* | 2017 | Net | UK | NM | Sacramento | Upper Karr Campground | 32.882 | -105.775 | 2760 | Abdomen | Yes |
| MM_BBSL1059325 | *Bombus huntii* | 2017 | Net | MM | NM | Magdalena | South Baldy | 33.991 | -107.184 | 2979 | Abdomen | No |
| HM_20453 | *Bombus huntii* | 2018 | Net | HM | AZ | White | Hannagan | 33.632 | -109.325 | 2793 | Gut | Yes |
| HM_20457 | *Bombus huntii* | 2018 | Net | HM | AZ | White | Hannagan | 33.632 | -109.325 | 2793 | Gut | Yes |
| HM_20458 | *Bombus huntii* | 2018 | Net | HM | AZ | White | Hannagan | 33.632 | -109.325 | 2793 | Gut | Yes |
| HM_20467 | *Bombus huntii* | 2018 | Net | HM | AZ | White | Hannagan | 33.632 | -109.325 | 2793 | Gut | Yes |
| HM_20468 | *Bombus huntii* | 2018 | Net | HM | AZ | White | Hannagan | 33.632 | -109.325 | 2793 | Gut | Yes |
| HM_20486 | *Bombus huntii* | 2018 | Net | HM | AZ | White | Hannagan | 33.632 | -109.325 | 2793 | Gut | Yes |
| HM_20490 | *Bombus huntii* | 2018 | Net | HM | AZ | White | Hannagan | 33.632 | -109.325 | 2793 | Gut | Yes |
| MM_20618 | *Bombus huntii* | 2018 | Net | MM | NM | Magdalena | South Baldy | 33.991 | -107.184 | 2979 | Gut | Yes |
| SC_20705 | *Bombus huntii* | 2018 | Net | SC | NM | Sandias | Kiwanis | 35.206 | -106.443 | 3091 | Gut | Yes |
| SC_20706 | *Bombus huntii* | 2018 | Net | SC | NM | Sandias | Kiwanis | 35.206 | -106.443 | 3091 | Gut | Yes |
| SC_20714 | *Bombus huntii* | 2018 | Net | SC | NM | Sandias | Kiwanis | 35.206 | -106.443 | 3091 | Gut | Yes |
| SC_20718 | *Bombus huntii* | 2018 | Net | SC | NM | Sandias | Kiwanis | 35.206 | -106.443 | 3091 | Gut | Yes |
| SC_20726 | *Bombus huntii* | 2018 | Net | SC | NM | Sandias | Kiwanis | 35.206 | -106.443 | 3091 | Gut | Yes |
| SC_20729 | *Bombus huntii* | 2018 | Net | SC | NM | Sandias | Kiwanis | 35.206 | -106.443 | 3091 | Gut | Yes |
| SC_20735 | *Bombus huntii* | 2018 | Net | SC | NM | Sandias | Kiwanis | 35.206 | -106.443 | 3091 | Gut | Yes |
| SC_20736 | *Bombus huntii* | 2018 | Net | SC | NM | Sandias | Kiwanis | 35.206 | -106.443 | 3091 | Gut | Yes |
| SC_20743 | *Bombus huntii* | 2018 | Net | SC | NM | Sandias | Kiwanis | 35.206 | -106.443 | 3091 | Gut | Yes |
| MM_20619 | *Bombus huntii* | 2018 | Net | MM | NM | Magdalena | South Baldy | 33.991 | -107.184 | 2979 | Gut | No* |
| SC_20704 | *Bombus huntii* | 2018 | Net | SC | NM | Sandias | Kiwanis | 35.206 | -106.443 | 3091 | Gut | No* |
| HM_20493 | *Bombus huntii* | 2018 | Net | HM | AZ | White | Hannagan | 33.632 | -109.325 | 2793 | Gut | No |
| MM_20599 | *Bombus huntii* | 2018 | Net | MM | NM | Magdalena | South Baldy | 33.991 | -107.184 | 2979 | Gut | No |
| SC_20712 | *Bombus huntii* | 2018 | Net | SC | NM | Sandias | Kiwanis | 35.206 | -106.443 | 3091 | Gut | No |
| HM_33417 | *Bombus huntii* | 2021 | Net | HM | AZ | White | Hannagan | 33.632 | -109.325 | 2793 | Gut | Yes |
| HM_33436 | *Bombus huntii* | 2021 | Net | HM | AZ | White | Hannagan | 33.632 | -109.325 | 2793 | Gut | Yes |
| HM_33439 | *Bombus huntii* | 2021 | Net | HM | AZ | White | Hannagan | 33.632 | -109.325 | 2793 | Gut | Yes |
| HM_33552 | *Bombus huntii* | 2021 | Net | HM | AZ | White | Hannagan | 33.632 | -109.325 | 2793 | Gut | Yes |
| HM_33570 | *Bombus huntii* | 2021 | Net | HM | AZ | White | Hannagan | 33.632 | -109.325 | 2793 | Gut | Yes |
| SC_30736 | *Bombus huntii* | 2021 | Net | SC | NM | Sandias | Kiwanis | 35.206 | -106.443 | 3091 | Gut | Yes |
| SC_30737 | *Bombus huntii* | 2021 | Net | SC | NM | Sandias | Kiwanis | 35.206 | -106.443 | 3091 | Gut | Yes |
| SC_30746 | *Bombus huntii* | 2021 | Net | SC | NM | Sandias | Kiwanis | 35.206 | -106.443 | 3091 | Gut | Yes |
| SC_30758 | *Bombus huntii* | 2021 | Net | SC | NM | Sandias | Kiwanis | 35.206 | -106.443 | 3091 | Gut | Yes |
| SC_30760 | *Bombus huntii* | 2021 | Net | SC | NM | Sandias | Kiwanis | 35.206 | -106.443 | 3091 | Gut | Yes |
| SC_30768 | *Bombus huntii* | 2021 | Net | SC | NM | Sandias | Kiwanis | 35.206 | -106.443 | 3091 | Gut | Yes |
| SC_30769 | *Bombus huntii* | 2021 | Net | SC | NM | Sandias | Kiwanis | 35.206 | -106.443 | 3091 | Gut | Yes |
| SC_31847 | *Bombus huntii* | 2021 | Net | SC | NM | Sandias | Kiwanis | 35.206 | -106.443 | 3091 | Gut | Yes |
| SC_32188 | *Bombus huntii* | 2021 | Net | SC | NM | Sandias | Kiwanis | 35.206 | -106.443 | 3091 | Gut | Yes |
| SC_32209 | *Bombus huntii* | 2021 | Net | SC | NM | Sandias | Kiwanis | 35.206 | -106.443 | 3091 | Gut | Yes |
| SC_32254 | *Bombus huntii* | 2021 | Net | SC | NM | Sandias | Kiwanis | 35.206 | -106.443 | 3091 | Gut | Yes |
| SM_31504 | *Bombus huntii* | 2021 | Net | SM | NM | San Mateo | La Mosca Lookout Tower | 35.248 | -107.599 | 3063 | Abdomen | Yes |
| SM_31507 | *Bombus huntii* | 2021 | Net | SM | NM | San Mateo | La Mosca Lookout Tower | 35.248 | -107.599 | 3063 | Abdomen | Yes |
| SM_31703 | *Bombus huntii* | 2021 | Net | SM | NM | San Mateo | La Mosca Lookout Tower | 35.248 | -107.599 | 3063 | Gut | Yes |
| HM_33422 | *Bombus huntii* | 2021 | Net | HM | AZ | White | Hannagan | 33.632 | -109.325 | 2793 | Gut | No* |
| HM_37620 | *Bombus huntii* | 2022 | Net | HM | AZ | White | Hannagan | 33.632 | -109.325 | 2793 | Abdomen | Yes |
| HM_37621 | *Bombus huntii* | 2022 | Net | HM | AZ | White | Hannagan | 33.632 | -109.325 | 2793 | Abdomen | Yes |
| MM_36492 | *Bombus huntii* | 2022 | Net | MM | NM | Magdalena | South Baldy | 33.991 | -107.184 | 2979 | Abdomen | Yes |
| MM_37072 | *Bombus huntii* | 2022 | Net | MM | NM | Magdalena | South Baldy | 33.991 | -107.184 | 2979 | Abdomen | Yes |
| MM_37085 | *Bombus huntii* | 2022 | Net | MM | NM | Magdalena | South Baldy | 33.991 | -107.184 | 2979 | Abdomen | Yes |
| MM_37109 | *Bombus huntii* | 2022 | Net | MM | NM | Magdalena | South Baldy | 33.991 | -107.184 | 2979 | Abdomen | Yes |
| SC_36445 | *Bombus huntii* | 2022 | Net | SC | NM | Sandias | Kiwanis | 35.206 | -106.443 | 3091 | Abdomen | Yes |
| SC_36614 | *Bombus huntii* | 2022 | Net | SC | NM | Sandias | Kiwanis | 35.206 | -106.443 | 3091 | Abdomen | Yes |
| SM_37174 | *Bombus huntii* | 2022 | Net | SM | NM | San Mateo | La Mosca Lookout Tower | 35.248 | -107.599 | 3063 | Abdomen | Yes |
| SM_37217 | *Bombus huntii* | 2022 | Net | SM | NM | San Mateo | La Mosca Lookout Tower | 35.248 | -107.599 | 3063 | Abdomen | Yes |
| SM_37338 | *Bombus huntii* | 2022 | Net | SM | NM | San Mateo | La Mosca Lookout Tower | 35.248 | -107.599 | 3063 | Abdomen | Yes |
| SM_37394 | *Bombus huntii* | 2022 | Net | SM | NM | San Mateo | La Mosca Lookout Tower | 35.248 | -107.599 | 3063 | Abdomen | Yes |
| HM_37623 | *Bombus huntii* | 2022 | Net | HM | AZ | White | Hannagan | 33.632 | -109.325 | 2793 | Abdomen | No |
| HM_37634 | *Bombus huntii* | 2022 | Net | HM | AZ | White | Hannagan | 33.632 | -109.325 | 2793 | Abdomen | No |
| HM_37636 | *Bombus huntii* | 2022 | Net | HM | AZ | White | Hannagan | 33.632 | -109.325 | 2793 | Abdomen | No |
| MM_37086 | *Bombus huntii* | 2022 | Net | MM | NM | Magdalena | South Baldy | 33.991 | -107.184 | 2979 | Abdomen | No |
| SC_36440 | *Bombus huntii* | 2022 | Net | SC | NM | Sandias | Kiwanis | 35.206 | -106.443 | 3091 | Abdomen | No |
| SC_36441 | *Bombus huntii* | 2022 | Net | SC | NM | Sandias | Kiwanis | 35.206 | -106.443 | 3091 | Abdomen | No |
| SC_36449 | *Bombus huntii* | 2022 | Net | SC | NM | Sandias | Kiwanis | 35.206 | -106.443 | 3091 | Abdomen | No |
| SM_37516 | *Bombus huntii* | 2022 | Net | SM | NM | San Mateo | La Mosca Lookout Tower | 35.248 | -107.599 | 3063 | Abdomen | No |

Table S2. Individual pairs with relatedness score (𝜙_𝑖𝑗_) greater than 0.2, indicating kinship. The samples removed from final analyses are indicated. We found one case in *B. centralis* where one individual was related to two individuals, but the other individuals were not related to each other. We removed this “repeated” individual from the dataset as indicated below.

|  | **Individual 1** | | | **Individual 2** | | |  |  |
| --- | --- | --- | --- | --- | --- | --- | --- | --- |
| **Species** | **Sample ID** | **Site** | **Collection Year** | **Sample ID** | **Site** | **Collection Year** | **Relatedness Score (**𝜙_𝑖𝑗_**)** | **Sample Removed** |
| *B. centralis* | HM_20489 | HM | 2018 | HM_20502 | HM | 2018 | 0.211885 | HM_20502 |
| *B. centralis* | HM_20494 | HM | 2018 | HM_20502 | HM | 2018 | 0.260242 | HM_20502 |
| *B. huntii* | MM_20618 | MM | 2018 | MM_20619 | MM | 2018 | 0.217912 | MM_20619 |
| *B. huntii* | SC_20704 | SC | 2018 | SC_20714 | SC | 2018 | 0.316008 | SC_20704 |
| *B. huntii* | HM_33422 | HM | 2021 | HM_33436 | HM | 2021 | 0.238773 | HM_33422 |

Table S3. Summary of global population genomics metrics calculated by Hierfstat for *Bombus centralis* and *B. huntii*.

| **Metric** | ***B. centralis*** | ***B. huntii*** |
| --- | --- | --- |
| Observed heterozygosity (H_O_) | 0.16 | 0.16 |
| Expected heterozygosity within populations (H_S_) | 0.19 | 0.27 |
| Total heterozygosity (H_T_) | 0.28 | 0.28 |
| Corrected Ht (H_TP_) | 0.3 | 0.28 |
| Genetic differentiation between populations (D_ST_) | 0.09 | 0.01 |
| Corrected Dst (D_STP_) | 0.11 | 0.01 |
| Fixation index (F_ST_) | 0.33 | 0.04 |
| Corrected Fixation index (F_STP_) | 0.37 | 0.05 |
| Inbreeding coefficient (F_IS_) | 0.16 | 0.39 |
| Genetic differentiation between populations (using allele frequencies) (D_EST_) | 0.13 | 0.02 |

Table S4. Summary of *Bombus centralis* and *B. huntii* expected heterozygosity among populations (H_s_), observed heterozygosity (H_O_), inbreeding coefficient (F_IS_) averaged across loci at the population level, and within-population Spearman’s correlation tests between H_O_ or F_IS_ and loci missingness. The number of loci used for either test is provided and matches criteria in Hierfstat, where H_O_ and H_s_ are estimated using all loci present in the population, and F_IS_ estimation excludes monomorphic loci. Loci missingness is estimated separately for each loci set to calculate Spearman’s ρ. We consider Spearman’s ρ between -1 and 1 to be negligible, and -2 < ρ < -1 or 1 < ρ < 2 to be weak.

|  | | | | **Spearman's Correlation - H_O_** | | | | | **Spearman's Correlation - F_IS_** | | | | |
| --- | --- | --- | --- | --- | --- | --- | --- | --- | --- | --- | --- | --- | --- |
| **Species** | **Pop.** | **Indiv. #** | **H_S_** | **Loci #** | **H_O_** | **Missing** | **ρ** | **P Value** | **Loci #** | **F_IS_** | **Missing** | **ρ** | **P Value** |
| *Bombus centralis* | HM | 27 | 0.2459 | 5064 | 0.1979 | 21.54% | -0.071 | 4.72E-07 | 4114 | 0.1629 | 21.63% | 0.061 | 9.41E-05 |
|  | MM | 14 | 0.1862 | 5064 | 0.1635 | 16.18% | 0.025 | 0.072 | 2996 | 0.0886 | 16.39% | -0.017 | 0.364 |
|  | SC | 17 | 0.1526 | 5058 | 0.1335 | 16.93% | 0.021 | 0.143 | 2636 | 0.0903 | 17.21% | -0.022 | 0.250 |
|  | SM | 25 | 0.2113 | 5064 | 0.1716 | 22.50% | -0.003 | 0.824 | 3571 | 0.1486 | 22.77% | 0.026 | 0.121 |
|  | JC | 21 | 0.2387 | 5064 | 0.1876 | 11.87% | -0.050 | 4.00E-04 | 4114 | 0.1843 | 11.87% | 0.068 | 1.33E-05 |
|  | PL | 16 | 0.0860 | 5044 | 0.0936 | 17.48% | 0.111 | 2.35E-15 | 1645 | -0.0665 | 19.16% | -0.018 | 0.462 |
| *Bombus huntii* | HM | 19 | 0.2687 | 3761 | 0.1879 | 21.46% | 0.042 | 0.010 | 3449 | 0.2674 | 21.33% | -0.038 | 0.026 |
|  | MM | 11 | 0.2851 | 3761 | 0.1616 | 48.03% | -0.024 | 0.137 | 2720 | 0.3558 | 45.86% | -0.060 | 0.002 |
|  | SC | 30 | 0.2701 | 3761 | 0.2166 | 18.44% | -0.015 | 0.357 | 3678 | 0.1795 | 18.39% | -0.030 | 0.067 |
|  | SM | 7 | 0.2732 | 3761 | 0.1762 | 21.77% | -0.049 | 0.002 | 2642 | 0.2842 | 20.15% | -0.009 | 0.627 |
|  | SS | 5 | 0.2633 | 3275 | 0.1039 | 57.72% | -0.183 | 6.13E-26 | 929 | 0.4561 | 41.12% | 0.025 | 0.440 |
|  | UK | 5 | 0.2654 | 3606 | 0.1268 | 41.69% | -0.174 | 5.30E-26 | 1555 | 0.3812 | 31.18% | 0.032 | 0.212 |

Table S5: All pairwise F_ST_ values between populations for *Bombus centralis* and *B. huntii*. Pairwise F_ST_ was calculated using the Weir and Cockerham method. Confidence intervals were generated using 1,000 bootstraps.

| **Species** | **Population Pair** | **Pairwise F_ST_** | **Lower Limit** | **Upper Limit** |
| --- | --- | --- | --- | --- |
| *Bombus centralis* | HM - JC | 0.145308 | 0.137208 | 0.153421 |
|  | HM - MM | 0.167959 | 0.158098 | 0.177414 |
|  | JC - MM | 0.244435 | 0.232926 | 0.255715 |
|  | HM - PL | 0.447184 | 0.433880 | 0.460472 |
|  | JC - PL | 0.425012 | 0.411459 | 0.438516 |
|  | MM - PL | 0.576809 | 0.562846 | 0.590882 |
|  | HM - SC | 0.341567 | 0.328674 | 0.353669 |
|  | JC - SC | 0.300814 | 0.287974 | 0.312272 |
|  | MM - SC | 0.450811 | 0.436802 | 0.465368 |
|  | PL - SC | 0.631752 | 0.619110 | 0.645159 |
|  | HM - SM | 0.133379 | 0.125862 | 0.141149 |
|  | JC - SM | 0.214759 | 0.204510 | 0.225316 |
|  | MM - SM | 0.256244 | 0.244340 | 0.267992 |
|  | PL - SM | 0.515046 | 0.501187 | 0.529051 |
|  | SC - SM | 0.409996 | 0.395419 | 0.422805 |
| *Bombus huntii* | HM - MM | 0.014341 | 0.008593 | 0.020034 |
|  | HM - SC | 0.011858 | 0.009560 | 0.014121 |
|  | MM - SC | 0.019824 | 0.014661 | 0.025190 |
|  | HM - SM | 0.020411 | 0.015097 | 0.025604 |
|  | MM - SM | 0.010762 | 0.001672 | 0.019881 |
|  | SC - SM | 0.022844 | 0.018138 | 0.028188 |
|  | HM - SS | 0.088192 | 0.074777 | 0.103102 |
|  | MM - SS | 0.048359 | 0.030797 | 0.067490 |
|  | SC - SS | 0.090054 | 0.077185 | 0.104106 |
|  | SM - SS | 0.071753 | 0.053079 | 0.090953 |
|  | HM - UK | 0.063857 | 0.052649 | 0.076955 |
|  | MM - UK | 0.036561 | 0.020671 | 0.052258 |
|  | SC - UK | 0.063016 | 0.052231 | 0.075307 |
|  | SM - UK | 0.039953 | 0.025177 | 0.054271 |
|  | SS - UK | 0.031981 | 0.006925 | 0.056828 |

Table S6: Total private allele counts and the estimated number of private alleles per individual for each population of *Bombus centralis* and *B. huntii*.

| **Species** | **Population** | **Total Private Alleles** | **Private Alleles per Individual** |
| --- | --- | --- | --- |
| *B. centralis* | HM | 333 | 12.33 |
|  | JC | 1427 | 67.95 |
|  | MM | 253 | 18.07 |
|  | PL | 3261 | 203.81 |
|  | SC | 2281 | 134.18 |
|  | SM | 659 | 26.36 |
| *B. huntii* | HM | 33 | 1.74 |
|  | MM | 0 | 0 |
|  | SC | 183 | 6.10 |
|  | SM | 0 | 0 |
|  | SS | 0 | 0 |
|  | UK | 0 | 0 |

Table S7: Final optimized resistance distance matrices generated by resistanceGA. Resolution of the input data is indicated under Resistance Type.

| **Species** | **Resistance Type** | **Transformation** | **Shape** | **Resistance Matrix** | | | | | | |
| --- | --- | --- | --- | --- | --- | --- | --- | --- | --- | --- |
| *B. centralis* | Land Cover  (300 m) | --- | --- |  | HM | JC | MM | PL | SC | SM |
|  |  |  |  | HM | NA | 21.61180428 | 15.35145824 | 119.8312253 | 27.16711283 | 12.79950606 |
|  |  |  |  | JC | 21.61180428 | NA | 29.8890081 | 137.9527127 | 18.05699438 | 18.79974394 |
|  |  |  |  | MM | 15.35145824 | 29.8890081 | NA | 132.5044179 | 34.18574985 | 21.87340882 |
|  |  |  |  | PL | 119.8312253 | 137.9527127 | 132.5044179 | NA | 143.2701296 | 129.8279744 |
|  |  |  |  | SC | 27.16711283 | 18.05699438 | 34.18574985 | 143.2701296 | NA | 24.86850048 |
|  |  |  |  | SM | 12.79950606 | 18.79974394 | 21.87340882 | 129.8279744 | 24.86850048 | NA |
| *B. centralis* | Elevation  (300 m) | Inverse  Monomolecular | 0.742884374 |  | HM | JC | MM | PL | SC | SM |
|  |  |  |  | HM | NA | 2.822254265 | 2.375554663 | 7.942119578 | 3.145579549 | 2.258063445 |
|  |  |  |  | JC | 2.822254265 | NA | 2.700394908 | 9.082145783 | 2.070735153 | 2.045143542 |
|  |  |  |  | MM | 2.375554663 | 2.700394908 | NA | 8.608301859 | 2.939142237 | 2.22719473 |
|  |  |  |  | PL | 7.942119578 | 9.082145783 | 8.608301859 | NA | 9.388946609 | 8.581257178 |
|  |  |  |  | SC | 3.145579549 | 2.070735153 | 2.939142237 | 9.388946609 | NA | 2.380955239 |
|  |  |  |  | SM | 2.258063445 | 2.045143542 | 2.22719473 | 8.581257178 | 2.380955239 | NA |
| *B. centralis* | Temperature (1 km) | Inverse-Reverse  Monomolecular | 0.87488967 |  | HM | JC | MM | PL | SC | SM |
|  |  |  |  | HM | NA | 3.098713476 | 3.111638451 | 11.50493594 | 3.856972541 | 2.59605853 |
|  |  |  |  | JC | 3.098713476 | NA | 3.203134939 | 12.68470295 | 2.454562372 | 2.07595198 |
|  |  |  |  | MM | 3.111638451 | 3.203134939 | NA | 12.65052853 | 3.84019046 | 2.7953486 |
|  |  |  |  | PL | 11.50493594 | 12.68470295 | 12.65052853 | NA | 13.43007687 | 12.2396162 |
|  |  |  |  | SC | 3.856972541 | 2.454562372 | 3.84019046 | 13.43007687 | NA | 2.82040984 |
|  |  |  |  | SM | 2.59605853 | 2.07595198 | 2.7953486 | 12.2396162 | 2.82040984 | NA |
| *B. centralis* | Precipitation (1 km) | Inverse  Monomolecular | 0.501609168 |  | HM | JC | MM | PL | SC | SM |
|  |  |  |  | HM | NA | 4.009907545 | 4.483370359 | 2.602045253 | 4.513639633 | 3.97967948 |
|  |  |  |  | JC | 4.009907545 | NA | 5.367665568 | 5.53564084 | 2.251419604 | 3.678441832 |
|  |  |  |  | MM | 4.483370359 | 5.367665568 | NA | 6.099951781 | 5.724246932 | 5.690764735 |
|  |  |  |  | PL | 2.602045253 | 5.53564084 | 6.099951781 | NA | 6.047118246 | 5.590348792 |
|  |  |  |  | SC | 4.513639633 | 2.251419604 | 5.724246932 | 6.047118246 | NA | 4.106499383 |
|  |  |  |  | SM | 3.97967948 | 3.678441832 | 5.690764735 | 5.590348792 | 4.106499383 | NA |
| *B. huntii* | Land Cover  (300 m) | --- | --- |  | HM | MM | SC | SM | SS | UK |
|  |  |  |  | HM | NA | 2.321561252 | 2.558051472 | 2.279754249 | 3.257779597 | 3.215689263 |
|  |  |  |  | MM | 2.321561252 | NA | 2.336278734 | 2.16148809 | 2.944206425 | 2.898412593 |
|  |  |  |  | SC | 2.558051472 | 2.336278734 | NA | 2.185040258 | 2.982411061 | 2.935498485 |
|  |  |  |  | SM | 2.279754249 | 2.16148809 | 2.185040258 | NA | 3.052580829 | 3.007683542 |
|  |  |  |  | SS | 3.257779597 | 2.944206425 | 2.982411061 | 3.052580829 | NA | 1.616887732 |
|  |  |  |  | UK | 3.215689263 | 2.898412593 | 2.935498485 | 3.007683542 | 1.616887732 | NA |
| *B. huntii* | Elevation  (300 m) | Inverse  Monomolecular | 0.555376831 |  | HM | MM | SC | SM | SS | UK |
|  |  |  |  | HM | NA | 1.400908769 | 1.628639802 | 1.379434151 | 2.124948425 | 2.080984252 |
|  |  |  |  | MM | 1.400908769 | NA | 1.477615668 | 1.305716453 | 1.925742208 | 1.879741508 |
|  |  |  |  | SC | 1.628639802 | 1.477615668 | NA | 1.3020723 | 1.954080929 | 1.90480005 |
|  |  |  |  | SM | 1.379434151 | 1.305716453 | 1.3020723 | NA | 1.967741374 | 1.92083914 |
|  |  |  |  | SS | 2.124948425 | 1.925742208 | 1.954080929 | 1.967741374 | NA | 0.763212074 |
|  |  |  |  | UK | 2.080984252 | 1.879741508 | 1.90480005 | 1.92083914 | 0.763212074 | NA |
| *B. huntii* | Temperature (1 km) | Inverse-Reverse  Monomolecular | 0.742374008 |  | HM | MM | SC | SM | SS | UK |
|  |  |  |  | HM | NA | 1.826167201 | 2.200786042 | 1.61597432 | 3.311069634 | 3.225388426 |
|  |  |  |  | MM | 1.826167201 | NA | 2.125588908 | 1.658725435 | 3.12257588 | 3.035166937 |
|  |  |  |  | SC | 2.200786042 | 2.125588908 | NA | 1.653768732 | 3.086370209 | 2.995638229 |
|  |  |  |  | SM | 1.61597432 | 1.658725435 | 1.653768732 | NA | 2.97770923 | 2.889412702 |
|  |  |  |  | SS | 3.311069634 | 3.12257588 | 3.086370209 | 2.97770923 | NA | 0.743289548 |
|  |  |  |  | UK | 3.225388426 | 3.035166937 | 2.995638229 | 2.889412702 | 0.743289548 | NA |
| *B. huntii* | Precipitation (1 km) | Monomolecular | 2.337579961 |  | HM | MM | SC | SM | SS | UK |
|  |  |  |  | HM | NA | 707.7569335 | 764.7971572 | 757.8872392 | 804.428002 | 807.5956171 |
|  |  |  |  | MM | 707.7569335 | NA | 624.2929893 | 641.0259473 | 669.6313395 | 670.4621712 |
|  |  |  |  | SC | 764.7971572 | 624.2929893 | NA | 641.5410577 | 733.7602642 | 734.0991017 |
|  |  |  |  | SM | 757.8872392 | 641.0259473 | 641.5410577 | NA | 762.5404376 | 763.6850456 |
|  |  |  |  | SS | 804.428002 | 669.6313395 | 733.7602642 | 762.5404376 | NA | 487.6153164 |
|  |  |  |  | UK | 807.5956171 | 670.4621712 | 734.0991017 | 763.6850456 | 487.6153164 | NA |

## Table S8. Outputs of Generalized Least Squares (GLS) models with a Maximum Likelihood Population Effects (MLPE) correlation structure evaluating the effects of Euclidean distance, land cover resistance, elevation resistance, temperature resistance, and precipitation resistance on the pairwise F_ST_ of Bombus centralis and B. huntii. Input raster resolution, term, estimate, standard error, t value, p value, and the corrected Akaike information criterion (AIC) are indicated for each model.

| **Species** | **IBD/IBR** | **Resolution (m)** | **Term** | **Est.** | **Std. Error** | **T Value** | **P Value** | **AICc** |
| --- | --- | --- | --- | --- | --- | --- | --- | --- |
| *Bombus centralis* | Distance (IBD) | --- | Euclidean distance | 2.28E-04 | 0.0095 | 0.024 | 0.981 | -22.485 |
|  | Land cover (IBR) | 300 | Land cover resistance | 0.0023 | 6.51E-04 | 3.589 | 0.0033 | -30.349 |
|  | Elevation (IBR) | 300 | Elevation resistance | 0.036 | 0.012 | 3.063 | 0.0091 | -27.972 |
|  | Temperature (IBR) | 1,000 | Temperature resistance | 0.025 | 0.0078 | 3.248 | 0.0064 | -28.502 |
|  | Precipitation (IBR) | 1,000 | Precipitation resistance | 0.0054 | 0.0117 | 0.463 | 0.651 | -22.727 |
| *Bombus huntii* | Distance (IBD) | --- | Euclidean distance | 0.017 | 0.00503 | 3.290 | 0.0059 | -62.454 |
|  | Land cover (IBR) | 300 | Land cover resistance | 0.036 | 0.0071 | 5.074 | 2.00E-04 | -69.856 |
|  | Elevation (IBR) | 300 | Elevation resistance | 0.044 | 0.0089 | 4.136 | 3.00E-04 | -68.954 |
|  | Temperature (IBR) | 1,000 | Temperature resistance | 0.022 | 0.00432 | 5.049 | 0.0002 | -69.672 |
|  | Precipitation (IBR) | 1,000 | Precipitation resistance | 2.18E-04 | 4.96E-05 | 4.387 | 7E-04 | -64.705 |

Table S9: Resistance values assigned to each land cover class by resistanceGA for *Bombus centralis* and *B. huntii*. Resistance values range from 1 to 1,000, with lower values indicating lower resistance to gene flow.

| **Land Cover Class** | ***Bombus centralis*** | ***Bombus huntii*** |
| --- | --- | --- |
| Temperate needle leaf forest | 1.00 | 2.20 |
| Tropical evergreen forest | 537.12 | 737.85 |
| Tropical deciduous forest | 497.23 | 704.13 |
| Temperate deciduous forest | 15.80 | 950.42 |
| Mixed forest | 326.95 | 621.63 |
| Tropical shrubland | 984.36 | 3.82 |
| Temperate shrubland | 104.91 | 1.00 |
| Tropical grassland | 963.30 | 1000.00 |
| Temperate grassland | 598.99 | 981.25 |
| Wetland | 586.62 | 217.76 |
| Cropland | 621.15 | 441.13 |
| Barren | 390.48 | 760.97 |
| Urban | 902.88 | 5.77 |
| Water | 536.35 | 201.56 |
